# The efflux pump SugE2 involved in protection of *Salmonella* 4,[5],12:i:- against quaternary ammonium salts and inhibition of virulence

**DOI:** 10.1371/journal.ppat.1012951

**Published:** 2025-03-18

**Authors:** Yuqi Tian, Yaya Wen, Xueying Wang, Youkun Zhang, Xilong Kang, Chuang Meng, Zhiming Pan, Xinan Jiao, Dan Gu

**Affiliations:** 1 Jiangsu Key Laboratory of Zoonosis, Yangzhou University, Yangzhou, Jiangsu, China; 2 Key Laboratory of Prevention and Control of Biological Hazard Factors (Animal Origin) for Agrifood Safety and Quality, Ministry of Agriculture of China, Yangzhou University, Yangzhou, Jiangsu, China; 3 Jiangsu Co-innovation Center for Prevention and Control of Important Animal Infectious Diseases and Zoonoses, Yangzhou University, Yangzhou, Jiangsu, China; 4 Joint International Research Laboratory of Agriculture and Agri-product Safety of the Ministry of Education, Yangzhou University, Yangzhou, Jiangsu, China; Universite Paris Descartes Faculte de Medecine, FRANCE

## Abstract

*Salmonella enterica* serovar 4,[5],12:i:-, a monophasic variant of *Salmonella* Typhimurium, has emerged as a common nontyphoidal *Salmonella* serotype to cause human foodborne disease, exhibiting antibiotic and multidrug resistance. In this study, we identified the isolates of *S*. 4,[5],12:i:- resistant to quaternary ammonium compounds (QACs) disinfectants, displaying elevated minimum inhibitory concentration (MIC) values (200 μg/mL) in Mueller-Hinton (MH) broth. The efflux pump SugE1 and SugE2 could be induced by didecyldimethylammonium bromide (DDAB) and found to be indispensable for *S*. 4,[5],12:i:- resistance to DDAB. The Hoechst 33342 dye accumulation and reduced ethidium bromide efflux in Δ*sugE1*, Δ*sugE2* and Δ*sugE1*Δ*sugE2* further confirmed the efflux function of SugE1 and SugE2. Moreover, DDAB inhibited the expression of *Salmonella* pathogenicity island 1 (SPI-1) to decrease the adhesion and invasion ability of *S*. 4,[5],12:i:- in IPEC-J2 cells, whereas the deletion of *sugE2* increased the adhesion and invasion ability. In an *in vivo* mice model, the virulence of Δ*sugE2* and Δ*sugE1*Δ*sugE2* mutant strains were enhanced and showed significantly increased bacterial loads in the liver, spleen, and cecum. The Δ*sugE2* and Δ*sugE1*Δ*sugE2* mutant strains exhibited an enhanced ability to disrupt the intestinal barrier, leading to systemic infection. Finally, we discovered that intestinal extracts could induce *sugE1* and *sugE2* expression, subsequently suppressing SPI-1 expression through SugE2, mediating the *Salmonella* intestinal infection process. In conclusion, our findings provide the pivotal role of the SugE2 efflux pump in conferring resistance to DDAB disinfectants and influencing bacterial virulence in *S*. 4,[5],12:i:-.

## Author summary

The escalating global public health concern posed by multidrug-resistant *S*. 4,[5],12:i:- has garnered widespread attention. The mechanisms underlying the acquisition of quaternary ammonium compound tolerance by *S*. 4,[5],12:i:- remain unclear. Our investigations provide significant insights into the mechanisms of QAC resistance and virulence in *S*. 4,[5],12:i:-, focusing on the efflux pump SugE. Our findings highlight the pivotal role of the SugE efflux pump in conferring resistance to DDAB, a widely used class of disinfectants, and also in influencing the virulence of this bacterium. Notably, our results demonstrate that intestinal extract can induce *sugE2* expression and inhibit SPI-1 expression, which mediate the adhesion, invasion, and oral infection of *S*. 4,[5],12:i:- both *in vitro* and *in vivo*. Understanding the biological role of SugE in disinfectant resistance and virulence will aid in assessing the risks associated with targeting efflux pumps to combat *Salmonella* dissemination and infection.

## Introduction

Since the first report of *Salmonella* 4,[5],12:i:- in Europe in the late 1990s, this serotype has rapidly disseminated globally, emerging as one of the predominant *Salmonella* serotype in both human and animal infections over the past two decades [1,2]. *S.* 4,[5],12:i:- ranks among the top five *Salmonella* serotypes among clinical human isolates in the United States, Europe, and China [1–3]. Notably, pigs, broilers, and cattle have been identified as primary hosts of *S.* 4,[5],12:i:- [4,5]. This serotype is the second predominant isolated *Salmonella* serotype from patients with diarrhea in Guangdong, China [6]. Furthermore, since 2014, *S.* 4,[5],12:i:- has emerged as the predominant strain among pigs in the United States, highlighting its widespread presence in animal reservoir [7]. In China, pigs continue to serve as the primary host, further highlighting the critical role of animal reservoirs in the persistence and transmission of *S*. 4,[5],12:i:- [8]. The emergence of the *S.* 4,[5],12:i:- ASSuT clone in pig farms has raised significant concerns due to its multidrug resistance to ampicillin, streptomycin, sulfonamides, and tetracycline, thereby complicating the control of *Salmonella* in pig farms [9,10]. As part of comprehensive infection control strategies, disinfectants are pivotal in preventing bacterial outbreaks, particularly those caused by multidrug-resistant pathogens in livestock settings. However, research on the tolerance of *S*. 4,[5],12:i:- to disinfectants remains limited.

Quaternary ammonium compounds (QACs) are widely used as effective disinfectants in both animal farms and the food industry [11]. However, *Salmonella* has evolved various mechanisms to resist QAC disinfectants, including alterations in cell membrane structure and composition, biofilm formation, and the activation of efflux pumps [12–15]. In the presence of the efflux pump inhibitors reserpine and Carbonyl Cyanide m-Chlorophenylhydrazone (CCCP), adapted *Salmonella* strains returned to their parent MIC of benzalkonium chloride [16], suggesting that efflux pumps play a pivotal role in *Salmonella*’s ability to adapt to QAC disinfectants. Beyond the well-characterized AcrAB and TolC efflux pumps, recent attention has focused on the small multidrug resistance (SMR) family of efflux pumps. The SMR efflux pumps is distinct from other multidrug transporters due to its relatively short length (105–150 amino acids) and their location in the 3′ conserved region of class I integrons [17]. SMR pumps require proton exchange to facilitate drug efflux [17,18]. Notably, the suppressor of *groEL* mutation protein E (SugE) is a prominent protein subfamily within the SMR family, that enables bacteria to resist antimicrobial agents and quaternary cationic compounds [19,20].

Recent studies have revealed a strong correlation between efflux pumps and pathogen virulence. In *Escherichia coli*, AcrAB-TolC system stands out as a major efflux pump responsible for multidrug resistance and involved in the regulation of virulence [21,22]. This efflux pump is capable of expelling intracellular toxic bile salts, which helps the *E. coli* survival in the intestine [23]. Subsequently, similar roles of the AcrAB system have been observed in various species, including *Pseudomonas aeruginosa* [24,25], *Neisseria gonorrhoeae* [26], and *S*. Typhimurium [27]. Studies in *S*. Typhimurium have demonstrated that mutants deficient in AcrB exhibit reduced adhesion and invasion capabilities in human intestinal epithelial cells and macrophages [28]. Moreover, the efflux pump MacAB has been shown to play a pivotal role in the virulence of *S.* Typhimurium in mouse models [29]. RNA-seq analysis have further revealed that the absence of *acrB* in *S*. Typhimurium SL1344 leads to downregulation of genes associated with *Salmonella* pathogenicity island 1 (SPI-1), SPI-2, and PhoPQ [30]. These findings indicate that efflux pumps significantly impact pathogen virulence, in addition to their known role in antibiotic resistance.

Our previous studies have found that *Salmonella* strains isolated from pig farms using QACs as disinfectants exhibited an increasing trend in resistance to these disinfectants [31]. In this study, we collected *S*. 4,[5],12:i:- strains isolated from this pig farm to determine their MIC values. Subsequently, we employed whole genome sequencing (WGS) and RNA-seq to identify the genes responsible for the high tolerance to DDAB. We identified the efflux pumps SugE1 and SugE2 as essential for conferring tolerance to disinfectants in *S.* 4,[5],12:i:- ZC055. Furthermore, our research revealed that the efflux pump SugE2 also influences the virulence of *S.* 4,[5],12:i:- ZC055 both *in vitro* and *in vivo*. Our findings provide novel insights into the role of the SugE efflux pump in bacterial virulence.

## Results

### *S.* 4,[5],12:i:- exhibits resistance to DDAB

Several studies have demonstrated the tolerance or even resistance of *Salmonella* to biocides [32]. Therefore, we utilized 109 *S*. 4,[5],12:i:- strains isolated from a pig farm in Shanghai, China, spanning the years 2016 to 2019, where QACs are employed as disinfectants. We subsequently determined the MIC values of these isolates to DDAB. As shown in S1 Table, *S.* 4,[5],12:i:- isolates ZC050, ZC055, and ZC201 exhibited the highest MIC values at 100 μg/mL in M9 medium, and all these strains were isolated in 2018. Additionally, MIC values were determined in MH broth, revealing that the isolates ZC004, ZC050, ZC055, and ZC201 displayed the highest MIC values at 200 μg/mL, which further confirmed the resistance of *S.* 4,[5],12:i:- to DDAB. The ZC055 strain has completed WGS and was selected for further investigation.

**Fig 1:**
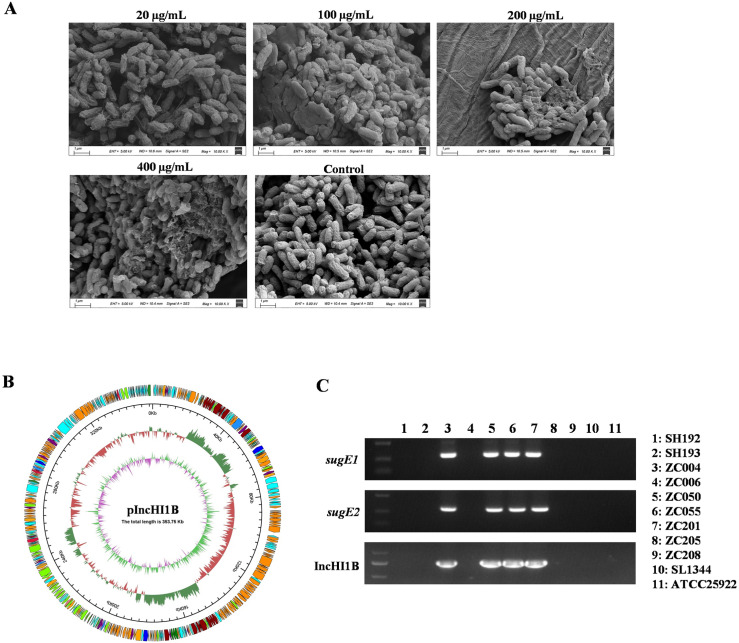
S. 4, [5], 12:i:- isolates demonstrating resistance to DDAB. (A) SEM images illustrate the morphological changes in the S. 4, [5], 12:i:- strain ZC055 following treatment with different concentrations of DDAB (0, 20, 100, 200, and 400 µg/mL). (B) A schematic representation of the IncHI1B plasmid map is provided. The outermost layer depicts COG functional annotation genes, with arrows in a clockwise direction indicating positive strand encoding. The subsequent layer displays plasmid sequence location coordinates. The third layer illustrates GC content, calculated using a 500 bp window and a 20 bp step size. Red areas indicate GC content lower than the average for the entire plasmid, while green areas denote higher GC content. The height of the peaks represents the magnitude of the difference from the average GC content. The innermost layer portrays GC skew, also calculated using a 500 bp window and a 20 bp step size. Pink areas signify lower G content compared to C, whereas light green areas indicate the opposite. (C) PCR was employed to detect the presence of *sugE1*, *sugE2*, and the IncHI1B plasmid in the S. 4, [5], 12:i:- isolates.

The morphology of *S.* 4,[5],12:i:- ZC055 treated with varying concentrations of DDAB was examined using scanning electron microscopy (SEM). In the control group, cells maintained a rod-like shape with intact structures. Upon treatment with DDAB, various alterations were observed in *Salmonella* cells (Fig 1A). At 20 µg/mL DDAB concentration, no significant changes were observed in cell structure compared to the control group. However, at 100 µg/mL DDAB, the bacterial cell membrane was disrupted, resulting in cavities on the cell surface. 200 µg/mL DDAB treatment led to disintegration of bacterial cells, whereas 400 µg/mL DDAB treatment resulted in even more pronounced dissolution of bacterial cells. SEM results indicated that elevated DDAB concentrations induced significant morphological changes in *S*. 4,[5],12:i:- cells.

### SugE1 and SugE2 are essential for *S.* 4,[5],12:i:- resistance to DDAB

**Fig 2:**
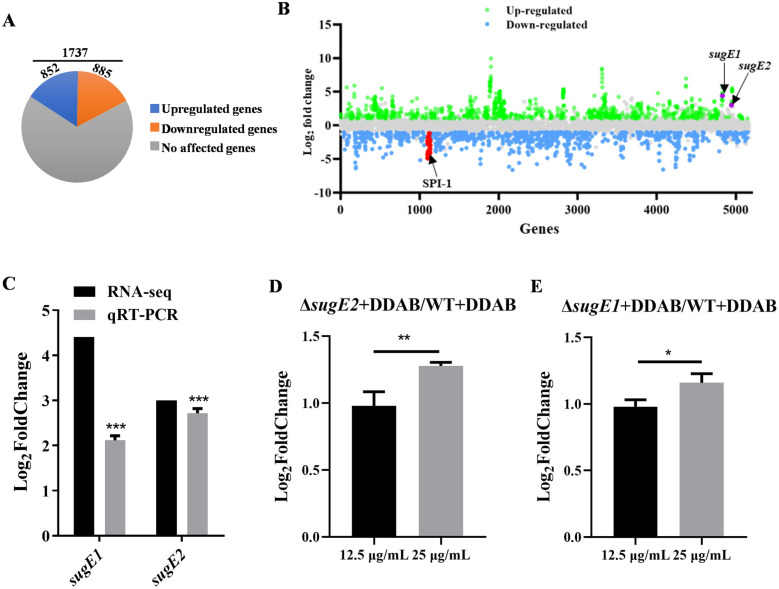
Transcriptome analysis of the S. 4, [5], 12:i:- ZC055 after treatment with 100 µg/mL DDAB. **(A)** Distribution of differentially expressed genes: blue represents upregulated genes after DDAB treatment, orange represents downregulated genes after DDAB treatment, and gray represents genes unaffected by DDAB. **(B)** Transcriptional landscape of ZC055 after treatment with DDAB compared to ZC055 cultured in LB medium. Significantly upregulated genes after DDAB treatment are marked in green, significantly downregulated genes are marked in blue, and gray indicates genes with no significant changes between the two groups. The *sugE1*, *sugE2*, and SPI-1 genes are marked in purple or red, respectively. **(C)** Validation of *sugE1* and *sugE2* gene expression using quantitative real-time PCR in accordance with the RNA-seq data. **(D)** Expression of the *sugE1* gene in Δ*sugE2* compared to WT after treatment with 12.5 µg/mL or 25 µg/mL DDAB. **(E)** Expression of the *sugE2* gene in Δ*sugE1* compared to WT after treatment with 12.5 µg/mL or 25 µg/mL DDAB.

WGS revealed the presence of two efflux pump genes, *sugE1* and *sugE2*, potentially facilitating bacterial resistance to disinfectants [17,19]. The *sugE1* and *sugE2* genes were located in the IncHI1B plasmid, which boasts a size of 353.75 kb and harbors a multitude of resistance genes, including *aac(6’)-Ib-cr*, *bla*_OXA-1_, *catB3*, *arr-3*, and *sul1* (Fig 1B). Subsequent PCR results showed that only the four isolates displaying high resistance to DDAB contained the *sugE1* and *sugE2* genes along with the IncHI1B plasmid (Fig 1C). Moreover, transcriptome analysis revealed significant upregulation of 852 genes and significant downregulation of 885 genes in *S*. 4,[5],12:i:- ZC055 when exposed to DDAB at concentrations of 100 µg/mL (1/2 MIC in LB medium) (Fig 2A), including ribosome, fatty acid biosynthesis, ABC transporters, flagellar assembly, and bacterial secretion system. Notably, *sugE1* and *sugE2* were among the significantly upregulated genes (Fig 2B), with their expression confirmed by qRT-PCR (Fig 2C). Additionally, we observed significant downregulation of the SPI-1 gene cluster, suggesting that DDAB may play a role in modulating the virulence of *S.* 4,[5],12:i:-.

To further validate the function of SugE1 and SugE2 in DDAB resistance, we introduced the IncHI1B plasmid into *S.* 4,[5],12:i:- FELB through electroporation and into *E. coli* C600 through conjugation. The MIC values of FFLB-pIncHI1B and C600-pIncHI1B were increased compared to those of the parental strains (Table 1). Deletion of the pIncHI1B plasmid in *S.* 4,[5],12:i:- ZC055 also led to a decrease in MIC values (Table 1). These results indicate the essential role of pIncHI1B in conferring resistance to DDAB in *S*. 4,[5],12:i:- ZC055. Subsequently, we generated single and double deletion mutant strains, Δ*sugE1*, Δ*sugE2*, and Δ*sugE1*Δ*sugE2*, to assess their contributions to DDAB resistance. The growth curves showed no differences among the wild-type (WT), Δ*sugE1*, Δ*sugE2*, and Δ*sugE1*Δ*sugE2* strains (S1 Fig). However, the MIC values of Δ*sugE1* and Δ*sugE2* were decreased in M9 minimal, MH broth or Luria-Bertani (LB) broth medium (Table 1). The complementation of these strains partially restored *Salmonella*’s resistance to DDAB. This indicates that SugE1 and SugE2 are indispensable for *S.* 4,[5],12:i:- ZC055 resistance to DDAB. The coexistence of these two genes in this strain and the low MIC values in Δ*sugE1*Δ*sugE2* suggest a potential compensatory effect between them. To explore this hypothesis, we compared the expression levels of *sugE1* or *sugE2* in Δ*sugE2* or Δ*sugE1* strains with WT under DDAB treatment at final concentrations of 12.5 μg/mL or 25 μg/mL. The results showed a significant increase in the expression level of *sugE1* in Δ*sugE2* compared to WT, and a more marked upregulation at higher DDAB concentrations (25 μg/mL) (Fig 2D). A similar trend was observed in the Δ*sugE1* strains, where *sugE2* expression was also significantly upregulated. (Fig 2E). Taken together, these results indicate that SugE1 and SugE2 play essential roles in DDAB resistance, and these two genes exhibit a compensatory effect.

**Table 1:** The MIC values of DDAB for the different strains.

Strains	M9 medium	MH broth	LB medium
	MIC (μg/mL)		
ZC055	100	200	200
ZC055ΔpIncHI1B	12.5	50	50
ZC055ΔpIncX1ΔpIncHI1B	12.5	50	50
C600	3.125	6.25	6.25
C600- pIncX1pIncHI1B	12.5	25	25
FFLB	6.25	12.5	12.5
FFLB-pIncHI1B	50	100	100
ZC055Δ*sugE1*	25	50	50
ZC055Δ*sugE2*	12.5	50	50
ZC055Δ*sugE1*Δ*sugE2*	12.5	25	25
ZC055Δ*sugE1*::*sugE1*	100	100	100
ZC055Δ*sugE2*::*sugE2*	100	100	100

### Unravelling the role of SugE1 and SugE2 efflux pumps via efflux abolishment

**Fig 3:**
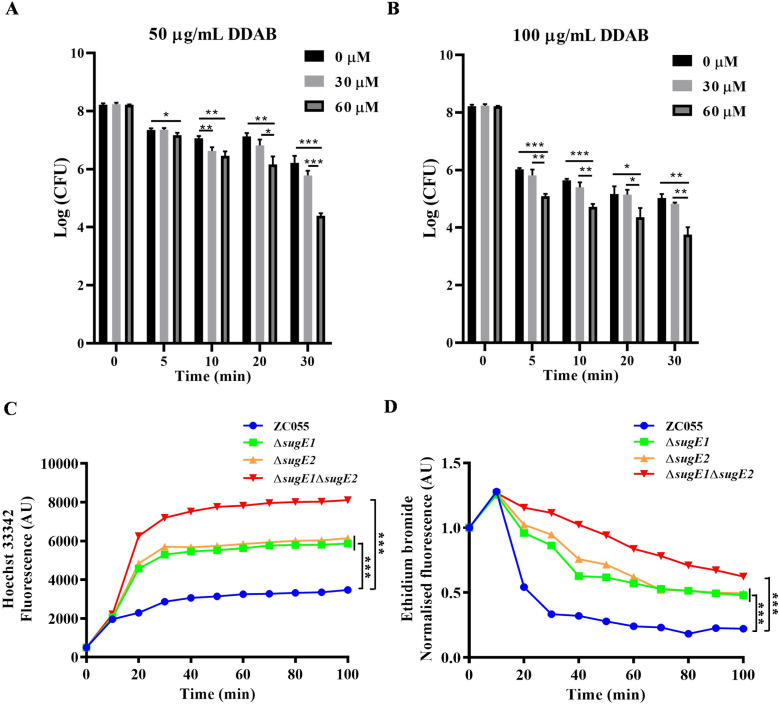
The efflux pump function of SugE1 and SugE2. (**A**-**B)** The viability of bacteria after treatment with 0, 30, and 60 μM CCCP, followed by the addition of 50 µg/mL DDAB (A) or 100 µg/mL DDAB **(B)**. **(C)** Accumulation kinetics and final fold change in accumulation of H33342 in ZC055, Δ*sugE1*, Δ*sugE2* and Δ*sugE1*Δ*sugE2*. **(D)** The efflux kinetics and final efflux fold change of ethidium bromide (ETBR) was examined in ZC055, Δ*sugE1*, Δ*sugE2* and Δ*sugE1*Δ*sugE2*.

We employed the proton-dependent efflux pump inhibitor CCCP to validate the efflux function of SugE1 and SugE2. We chose concentrations of 30 µM and 60 µM for CCCP to inhibit the activity of efflux pump (S2 Fig). After pre-treatment with CCCP, the clearance rate of 50 µg/mL DDAB on *S.* 4,[5],12:i:- ZC055 was significantly accelerated (Fig 3A). When concentration of DDAB was increased to 100 µg/mL, in the group of treatment with 60 µM CCCP, the clearance rate was significantly enhanced compared to the untreated group and 30 µM CCCP treatment group (Fig 3B). Thes results demonstrate that inhibition of efflux pump activity markedly enhances the antimicrobial activity of DDAB against *S.* 4,[5],12:i:- ZC055, highlighting the involvement of the efflux pump in bacterial resistance to DDAB.

Furthermore, we assessed the intracellular accumulation of Hoechst 33342 (H33342) and the efflux of ethidium bromide in ZC055, Δ*sugE1*, Δ*sugE2*, and Δ*sugE1*Δ*sugE2* strains. A time-dependent increase in fluorescence was observed as H33342 dye accumulated within bacterial cells until the system reached an equilibrium/steady state. The Δ*sugE1* and Δ*sugE2* strains achieved equilibrium faster than the ZC055 strain and exhibited significantly higher accumulation rates compared to the parental strain, while the double mutant strain Δ*sugE1*Δ*sugE2* displayed the highest H33342 dye accumulation (Fig 3C). To measure ethidium bromide efflux, we preloaded bacteria with the dye and then energized bacteria with glucose to initiate efflux, resulting in a gradual decrease in fluorescence over time. The Δ*sugE1* and Δ*sugE2* strains reached a steady state more rapidly than the ZC055 strain, with efflux rates approximately two-fold lower than those in the WT strain (Fig 3D). The Δ*sugE1*Δ*sugE2* strain also exhibited the highest accumulation of ethidium bromide. These results validated the efflux function of SugE1 and SugE2.

Subsequently, we constructed interaction models for DDAB with the SugE1 or SugE2 proteins, respectively. Within the SugE1 protein, DDAB engages in three alkyl/π-alkyl interactions with the amino acid residues Trp-22, Pro-23, and Phe-86, indicating its predominant binding to SugE1 through hydrophobic interactions. This is supported by a binding energy of -4.5 kcal/mol (S2A Fig). DDAB also forms a carbon-hydrogen bond with the amino acid residue Ile-66 and establishes four alkyl/π-alkyl interactions with Trp-3, Val-7, Ile-99, and Lys-102 in the SugE2 protein. This suggests a predominant binding to SugE2 through a combination of hydrophilic and hydrophobic interactions, with a binding energy of -4.8 kcal/mol (S2B Fig), which is notably slightly stronger than the interaction with SugE1. Taken together, these results indicate that the efflux pumps SugE1 and SugE2 may directly participate in the efflux of quaternary ammonium disinfectants.

### SugE2 inhibit the adhesion and invasion ability of *S*. 4,[5],12:i:-

**Fig 4:**
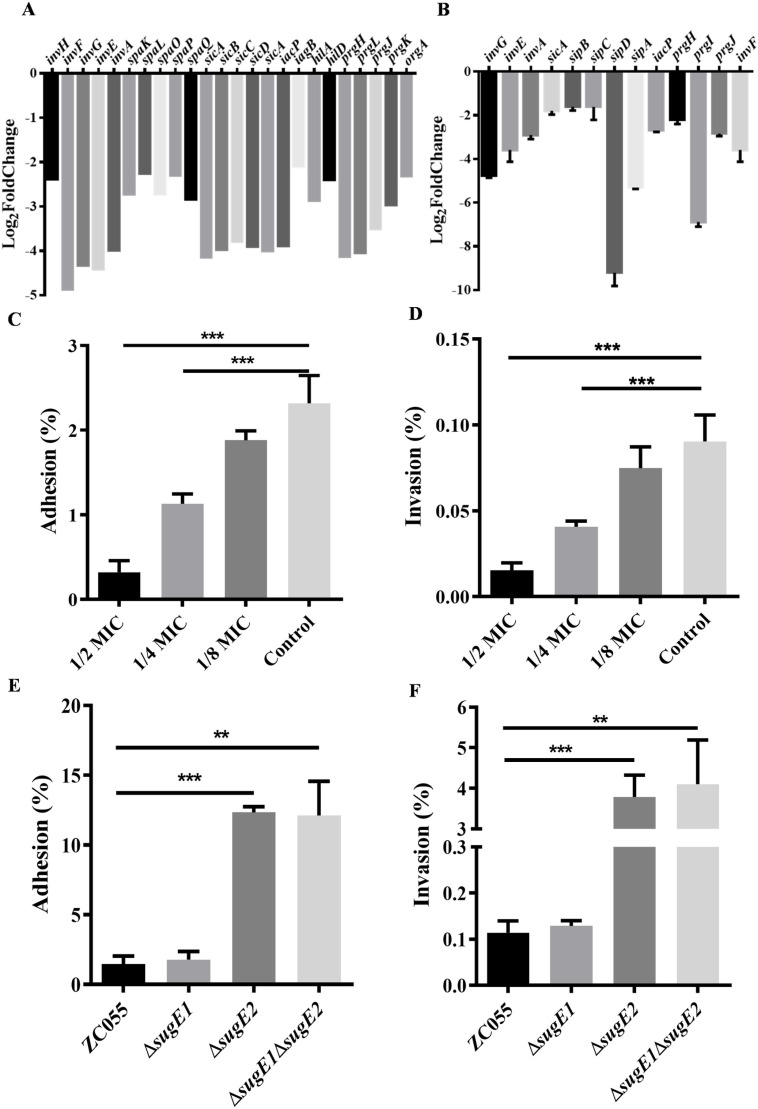
The adhesion and invasion ability of S. 4, [5], 12:i:- ZC055 and its derivate strains to IPEC-J2 cells. **(A)** The expression levels of T3SS1 in ZC055 treated with DDAB by RNA-seq. (B) qRT-PCR analysis of the expression levels of T3SS1 in ZC055 treated with 100 µg/mL DDAB. (**C-D)** The adhesion (C) and invasion (D) abilities of ZC055 treated with DDAB at concentrations of 1/2 MIC (100 µg/mL), 1/4 MIC (50 µg/mL), and 1/8 MIC (25 µg/mL) were compared to those of ZC055 cultured in LB medium in IPEC-J2 cells. (**E-F)** The infection assays were also performed using ZC055, Δ*sugE1*, Δ*sugE2* and Δ*sugE1*Δ*sugE2* in IPEC-J2 cells. The adhesion (E) and invasion (F) of Δ*sugE1*, Δ*sugE2*, and Δ*sugE1*Δ*sugE2* to IPEC-J2 cells were compared to the adhesion and invasion of ZC055.

RNA-seq data showed that DDAB inhibited the expression of SPI-1 (Figs 2B 4A), and then confirmed by RT-PCR (Fig 4B). Previous studies have reported the crucial role of *Salmonella* SPI-1 in the invasion of host cells, while the other studies have underscored the importance of efflux pumps in pathogen virulence [29,30,33–35]. Therefore, we assessed the adhesion and invasion capabilities of *S*. 4,[5],12:i:- ZC055 when exposed to varying concentrations of DDAB in porcine intestinal epithelial cells (IPEC-J2). Adhesion and invasion of the ZC055 strain to IPEC-J2 cells exhibited a significant reduction following treatment with DDAB at 1/2 and 1/4 MICs (Fig 4C and 4D). Furthermore, we explored the adhesion and invasion of *S*. 4,[5],12:i:- ZC055, Δ*sugE1*, Δ*sugE2* and Δ*sugE1*Δ*sugE2* strains in IPEC-J2 cells. The Δ*sugE2* and Δ*sugE1*Δ*sugE2* strains demonstrated heightened adhesion (Fig 4E) and invasion abilities (Fig 4F) compared to the WT strain, while ZC055Δ*sugE1* showed no significant differences compared to the WT strain. These results suggest that SugE2 is involved in regulating the adhesion and invasion abilities of *S*. 4,[5],12:i:- ZC055.

### SugE2 inhibits *S*. 4 ,[5],12:i:- virulence in a mice model

**Fig 5:**
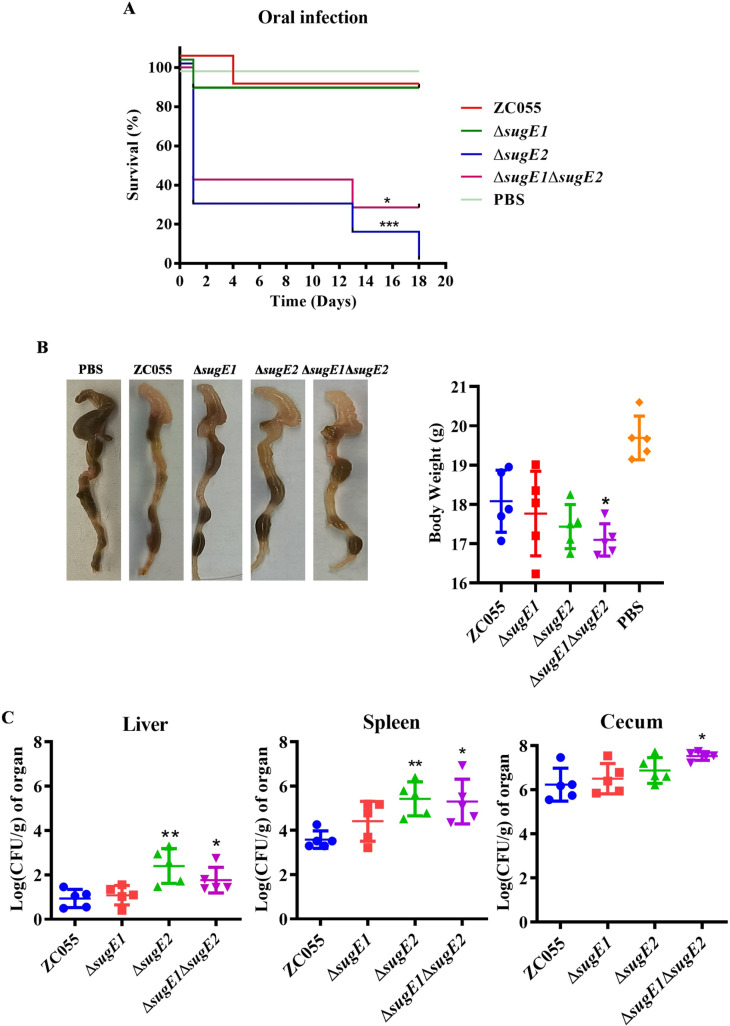
SugE2 inhibits the virulence of S. 4, [5], 12:i:- in C57BL/6 mice. ZC055, Δ*sugE1*, Δ*sugE2* and Δ*sugE1*Δ*sugE2* were orally infected in C57BL/6 mice. **(A)** Kaplan-Meier survival curves of mice for 18 days after infection. The *P* value was determined using a log rank (Mantel-Cox) test. **(B)** Representative images of cecal lesions on day 3 post-infection and comparison of the body weight of Δ*sugE1*, Δ*sugE2*, and Δ*sugE1*Δ*sugE2* infected mice with ZC055 infected mice on day 3 post-infection. **(C)** Bacterial load in the liver, spleen, and cecum of Δ*sugE1*, Δ*sugE2* and Δ*sugE1*Δ*sugE2* infected mice on day 3 post-infection. Organs were collected, homogenized, and the number of colony-forming units (CFU) was counted to quantify the bacterial load. The median values were plotted. A Mann-Whitney U test was used to compare the bacterial load in each organ of Δ*sugE1*, Δ*sugE2* and Δ*sugE1*Δ*sugE2* infected mice with ZC055 infected mice.

We further evaluated the virulence of ZC055, Δ*sugE1*, Δ*sugE2*, and Δ*sugE1*Δ*sugE2* strains, in orally infected C57BL/6 mice. Survival curve analysis revealed an accelerated mortality rate in Δ*sugE2* and Δ*sugE1*Δ*sugE2* strains, reaching 100% and 71%, respectively, whereas the mortality rates for the WT and Δ*sugE1* strains were lower, standing at 14% (Fig 5A). After three days of infection, the cecum exhibited swelling in Δ*sugE2*- and Δ*sugE1*Δ*sugE2*-infected mice, and there was also a significant weight loss observed in the Δ*sugE1*Δ*sugE2*-infected mice (Fig 5B). After three days of infection, the bacterial CFUs in the Δ*sugE2* and Δ*sugE1*Δ*sugE2*-infected mice were significantly higher than those in the WT- and Δ*sugE1*-infected mice within the liver and spleen (Fig 5C). Therefore, we hypothesized that the SugE2-deficient strain exhibited an enhanced capacity to breach the intestinal barrier, subsequently leading to systemic infection.

**Fig 6:**
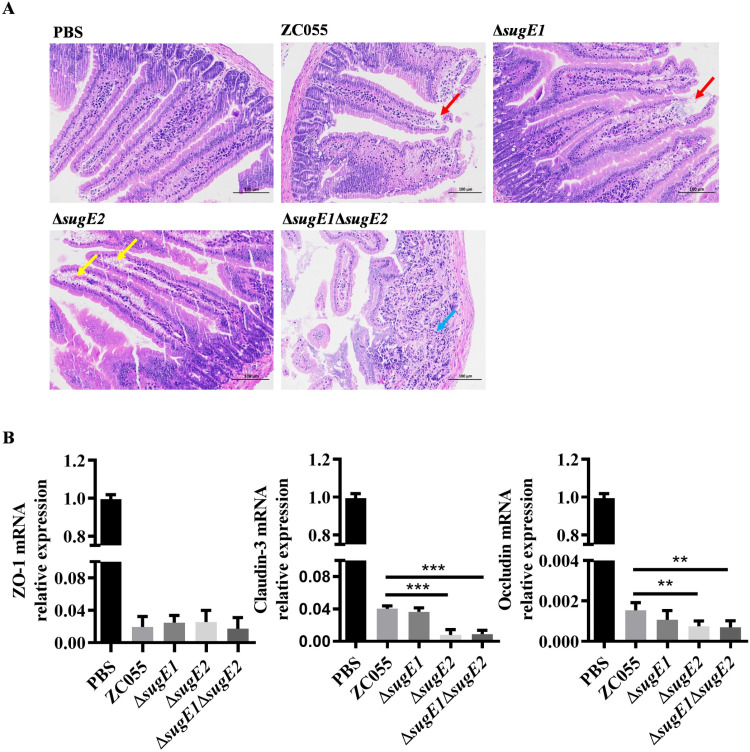
Intestinal pathological and expression levels of tight junction proteins in mice intestine after infection with S. 4, [5], 12:i:- ZC055 and its derivate strains. **(A)** Representative images of histopathological sections from the mouse intestine collected on day 3 post-infection. The red arrows denote exfoliation of epithelial cells in the intestinal villi. The yellow arrows denote edema of the intestinal villi with detachment of the epithelium from the lamina propria. The blue arrows denote lymphocytic infiltration (magnification is 200 ×). **(B)** Expression levels of tight junction proteins, including Zona Occludens-1 (ZO-1), Claudin-3, and Occludin, in the intestines of WT, Δs*ugE1*, Δ*sugE2* and Δ*sugE1*Δ*sugE2* infected mice on day 3 post-infection were quantified using qRT-PCR. The expression levels were compared to those of tight junction proteins in the intestine of ZC055 infected mice, with the results presented as fold changes relative to the control group (PBS).

To further validate this hypothesis, we collected the intestinal tract from the infected mice after a three-day and conducted a pathological analysis. Compared to the WT infected mice, both the Δ*sugE2* and Δ*sugE1*Δ*sugE2* infected mice induced more pronounced intestinal damage, including villous edema, epithelial sloughing from lamina propria, focal mucosal necrosis, goblet cell depletion, and lymphocytic infiltration (Fig 6A). Moreover, the expression levels of intestinal tight junction proteins, specifically claudin-3 and occludin, were significantly reduced in the Δ*sugE2* and Δ*sugE1*Δ*sugE2* strains compared to the WT strain, whereas the expression level of zonula occludens-1 (ZO-1) remained unaffected (Fig 6B).

### SugE2 inhibits the expression of T3SS1 to affect the adhesion and invasion abilities of ZC055 strain

**Fig 7:**
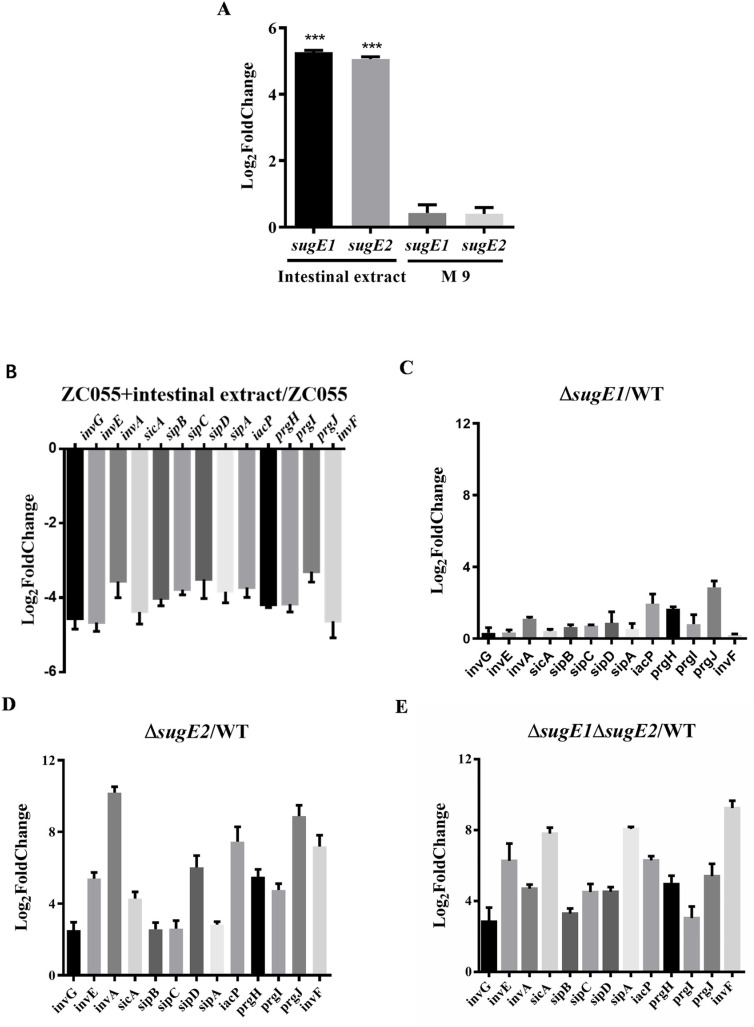
SugE2 senses the intestinal content to inhibit the expression of SPI-1. **(A)** The expression levels of the *sugE1* and *sugE2* genes in ZC055 cultured in LB medium supplemented with 50% mouse intestinal content or M9 medium. The results are presented as Log_2_foldchange relative to ZC055 cultured in LB medium. (B-**E)** The expression levels of the SPI-1 genes in ZC055 **(B)**, Δ*sugE1***(C)**, Δ*sugE2***(D)**, andΔ*sugE1*Δ*sugE2* (E) cultured in LB medium supplemented with 50% mouse intestinal content were quantified using qRT-PCR. The results are presented as Log_2_foldchange relative to WT cultured in LB medium.

RNA-seq indicated that DDAB induced the expression of *sugE1* and *sugE2*, whereas they inhibited the expression of SPI1-T3SS1 (Fig 2B). This prompted us to investigate the expression levels of *sugE1*, *sugE2*, and T3SS1 within the murine intestinal tract. In LB medium supplemented with intestinal extract, the expression levels of *sugE1* and *sugE2* were significantly increased, whereas no difference was observed in M9 medium (Fig 7A). Furthermore, we observed that the expression level of T3SS1 was significantly decreased in LB medium supplemented with intestinal extract (Fig 7B). These results indicate a significant upregulation in the expression levels of *sugE1* and *sugE2* within the intestinal tract, accompanied by a significant downregulation of T3SS1 expression. Moreover, we also determined the inhibition of T3SS1 under the conditions of intestinal tract was associated with SugE2. In the Δ*sugE1* strain, we observed no significant difference in the expression levels of T3SS1 compared to WT (Fig 7C). However, compared to WT, both the Δ*sugE2* (Fig 7D) and Δ*sugE1*Δ*sugE2* strains (Fig 7E) showed a significant increase in the expression levels of T3SS1.

**Fig 8:**
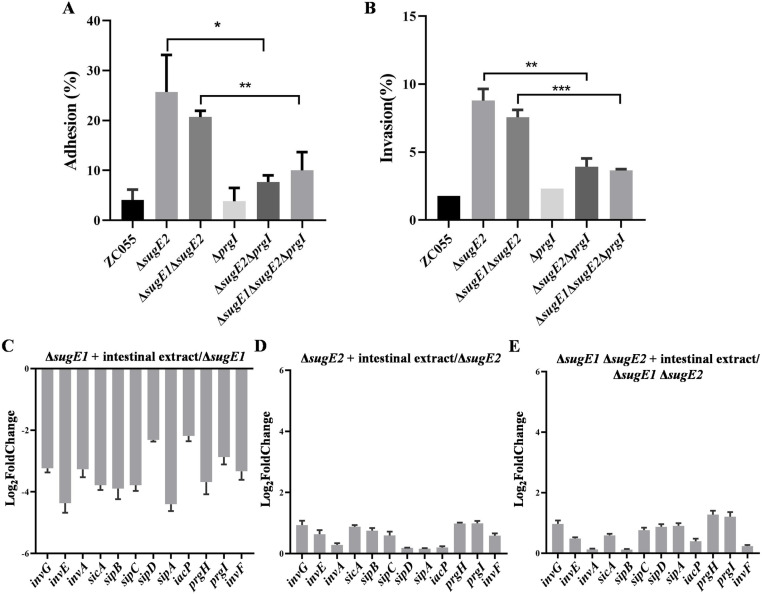
SugE2 inhibits the expression of SPI-1 to affect the adhesion and invasion of S. 4, [5], 12:i:- ZC055. The adhesion (A) and invasion (B) abilities of ZC055, Δ*sugE2*, Δ*sugE1*Δ*sugE2*, Δ*prgI*, Δ*prgI*Δ*sugE2*, and Δ*prgI*Δ*sugE1*Δ*sugE2* in IPEC-J2 cells. (**C-E)** The expression levels of the SPI-1 genes in Δ*sugE1* **(C)**, Δ*sugE2* (D) and Δ*sugE1*Δ*sugE2* (E) cultured in LB medium supplemented with 50% mouse intestinal content compared these strains cultured in LB medium.

To further confirm that the enhanced virulence observed after the deletion of *sugE2* gene is related to the increased expression of T3SS1, we constructed a deletion mutant of the *prgI* gene, a key component of T3SS1 essential for the assembly of the needle complex and the structural integrity and function of T3SS1 [36]. The WT, ∆*sugE2*, and ∆*sugE1*∆*sugE2* strains were used as the parents for this construction. We measured the adhesion and invasion abilities of WT, ∆*sugE2*, ∆*sugE1*∆*sugE2*, ∆*prgI*, ∆*sugE2*∆*prgI*, and ∆*sugE1*∆*sugE2*∆*prgI* in IPEC-J2 cells. Compared to the ∆*sugE2* and ∆*sugE1*∆*sugE2* strain, the adhesion (Fig 8A) and invasion (Fig 8B) abilities of ∆*sugE2*∆*prgI* and ∆*sugE1*∆*sugE2*∆*prgI* strains were significantly reduced. These results indicate that SugE2 inhibits the expression of SPI-1 to inhibit the adhesion and invasion of *S*. 4,[5],12:i:- ZC055. Furthermore, we explored whether intestinal extract affects SPI-1 expression through SugE2 by measuring SPI-1 levels in ∆*sugE1*, ∆*sugE2* and ∆*sugE1*∆*sugE2* after treatment with the intestinal extract. The expression of SPI-1 was significantly downregulated in the absence of *sugE1* (Fig 8C), while there was no difference in SPI-1 expression in ∆*sugE2* (Fig 8D) and ∆*sugE1*∆*sugE2* (Fig 8E) after treatment with the intestinal extract. Taken together, these results demonstrate that the intestinal contents induce the expression of SugE2, which subsequently leads to the transcriptional inhibition of T3SS1, thereby reducing the adhesion and invasion of *S*. 4,[5],12:i:- ZC055.

## Discussion

*S.* 4,[5],12:i:- represents a monophasic variant of *S*. Typhimurium, and it has emerged as the predominant serotype in human clinical samples, animals, and food products, posing a threat to public health security [1–3]. The *S.* 4,[5],12:i:- ZC055 variant belongs to the European clone, which is the most prevalent clone worldwide and harbors ASSuT-resistance genes, exhibiting robust resistance to various classes of antibiotics [37,38]. In a previous study, we showed that all *S.* 4,[5],12:i:- strains isolated from this pig farm exhibit multidrug resistance, with some exhibiting resistance to disinfectants [31]. In this study, we assessed the MIC values of *S.* 4,[5],12:i:- isolates against DDAB and found that the efflux pumps SugE1 and SugE2 play essential roles in *S.* 4,[5],12:i:- resistance to DDAB disinfectants. Furthermore, we report the involvement of SugE2 in the virulence of *S.* 4,[5],12:i:- (Fig 8).

Disinfectants are commonly employed in controlling pathogens throughout the food production chain; however, the dilution of disinfectants due to residual surface moisture during their application can lead to sublethal concentrations, thereby fostering the emergence of tolerant strains. Our research showed that the MIC values for *S.* 4,[5],12:i:- isolates in response to DDAB reached 200 μg/mL (S1 Table). *Salmonella* resistance to disinfectants is attributed to alterations in cell membrane structure and composition, biofilm formation, and the activation of efflux pumps [10,16,39,40]. Our investigations revealed that the *S.* 4,[5],12:i:- ZC055 strain lacked the ability to form biofilms, suggesting that its high resistance to QACs was not associated with biofilm formation. Subsequently, through WGS, we identified the presence of three *sugE* genes in S. 4,[5],12:i:- ZC055, which have previously been reported as essential for resistance to DDAB [41]. One *sugE* gene was located within the chromosome, while the other two, namely *sugE1* and *sugE2*, were situated in the IncHI1B plasmid (Fig 1B). Furthermore, RNA-seq demonstrated that DDAB could induce the expression of SugE1 and SugE2 (Fig 2B). The MIC values for Δ*sugE1* and Δ*sugE2* strains in response to DDAB decreased to 25 μg/mL and 12.5 μg/mL in M9 medium, underscoring the essential role of SugE1 and SugE2 in conferring resistance to DDAB disinfectants in *S.* 4,[5],12:i:- ZC055. The IncHI1B plasmid have historically been associated with *Klebsiella pneumoniae* and *E. coli*, harboring *bla*_CTX-M-15_, *bla*_NDM_, and quinolone-resistant determinant *qnrB1* [42,43]. In this study, we identified the emergence of IncHI1B plasmids in *S.* 4,[5],12:i:-, carrying *bla*_OXA-1_, *aac(6’)-Ib-cr*, *catB3*, *arr-3*, *sul1*, and three virulence genes (S2 Table). We demonstrated that the IncHI1B plasmid could be transferred to other *Salmonella* strains or *E. coli* through electroporation or conjugation, respectively. These findings indicate that the IncHI1B plasmid carrying multidrug resistance and virulence genes may pose a risk of dissemination.

Notably, we identified two SugE genes within the IncHI1B plasmid, with a 57% amino acid sequence identity between *sugE1* and *sugE2*. The MIC values for Δ*sugE1* and Δ*sugE2* against DDAB indicate that either SugE1 or SugE2 alone is sufficient for DDAB resistance, implying potential functional overlap between these two genes (Table 1). In *Listeria monocytogenes* EGD-e, two *sugE* genes are implicated in QAC resistance, although they are situated within the chromosome [44]. *P. aeruginosa* possesses three *sugE* genes [45], with the *sugE1* gene identified in plasmids across several members of Enterobacteriaceae [46], while the other homologs (*sugE2* and *sugE3*) are associated with the chromosomes [47]. In this study, we observed that the deletion of one *sugE* gene could significantly elevate the transcription of the other *sugE* gene in the presence of DDAB (Fig 2D and 2E). Similar findings have been reported in *L. monocytogenes* [44]. Additionally, the expression of *sugE* genes is subject to negative regulation by the transcriptional regulator SugR, which is situated upstream of *sugE* in *L. monocytogenes* [44]. Our findings showed that *sugE1* and *sugE2* are not expressed in LB medium without DDAB, but their induction occurs in the presence of DDAB. This indicates the existence of a mechanism in *Salmonella* that responds to DDAB signals to regulate the expression of *sugE*. However, a homologous protein of SugR in *S.* 4,[5],12:i:- ZC055 was not identified. Therefore, further research is required to elucidate this regulatory mechanism.

SMR proteins function as drug-metabolite transporters and confer resistance to various lipophilic compounds, such as QACs [17]. Current research on efflux pumps not only focuses on drug resistance, but also sheds light on their crucial role in the virulence of pathogens [28,48,49]. Efflux pumps such as AcrAB-TolC, MacAB, and MdsABC in *Salmonella* play essential roles in the infection process, as mutations in these efflux pump genes can attenuate the virulence of *Salmonella* [29,50–52]. However, research on the association between SMR and pathogenicity of pathogens, especially the SugE efflux pump, remain limited. Remarkably, our RNA-seq data indicated that DDAB can downregulate the expression of SP1-1, which is essential for *Salmonella*’s invasion into host cells, ultimately resulting in systemic infection in mice. Moreover, DDAB inhibit the adhesion and invasion of *S*. 4,[5],12:i:- ZC055 in IPEC-J2 cells (Fig 4C and 4D) possibly through the inhibition of SPI-1 transcripts when exposed to DDAB. Our RNA-seq data revealed that all genes located in SPI-1 were down-regulated in response to DDAB, including the key regulators in the SPI-1 regulatory hierarchy, such as *hilD*, *hilA*, and *invF*, which active the expression of T3SS1 associated genes within the SPI-1 locus. Previous studies have identified several key factors, including PhoP/PhoQ, OmpR/EnvZ, H-NS, and Lon, that regulate the expression of HilD, thereby influencing the expression of T3SS1 [53]. Our RNA-seq data also showed that the expression of *lon* was significantly increased upon treatment with DDAB. Another study in *S*. Typhimurium also found that treatment with disinfectant benzalkonium chloride activates Lon-mediated degradation of HilD, thereby downregulating the expression of the SPI-1 gene cluster [54]. Therefore, we speculate that the DDAB may stimulates Lon-mediated HilD proteolysis, leading to the downregulation of the expression of all SPI-1 genes.

In addition to the downregulation of SPI-1 expression by DDAB, we also observed an upregulation in the transcription of *sugE1* and *sugE2* genes in *S*. 4,[5],12:i:- ZC055 upon DDAB treatment. Consequently, we sought to investigate the potential association between SugE1/2 and the virulence of *S*. 4,[5],12:i:-. We found that deletion of the *sugE2* gene enhanced the adhesion and invasion capabilities of *S*. 4,[5],12:i:- in IPEC-J2 cells (Fig 4E and 4F) without affecting cytotoxicity and motility (S5 Fig). In a mice model, the *sugE2* mutant of *S*. 4,[5],12:i:- ZC055 exhibited increased virulence, leading to higher bacterial loads in the spleen, liver, and cecum compared to mice infected with the WT strain (Fig 5). In contrast, prior research on *Acinetobacter baumannii* has revealed that the deletion of the SMR-type efflux pump A1S_0710 reduces bacterial motility and virulence [55]. However, in the case of *Riemerella anatipestifer*, the deletion of the SMR-type efflux pump RanQ does not affect its virulence, adhesion, invasion, or biofilm formation [56]. Therefore, our study illuminates that SugE2 inhibits the adhesion, invasion, and virulence of *Salmonella*, highlighting the varying effects of different SMR-type efflux pumps on pathogen virulence.

The mutant of *S*. Typhimurium’s efflux pump, AcrB, exhibited reduced virulence and lower bacterial loads in both spleen and liver following oral and intraperitoneal infections [30]. However, our study revealed a significant increase in virulence of the Δ*sugE2* and Δ*sugE1*Δ*sugE2* strains during oral infections, whereas no significant difference in virulence was observed between the WT and mutant strains in intraperitoneal infections (Figs 5A and S3). Furthermore, we measured the growth abilities of WT, ∆*sugE1*, ∆*sugE2*, and ∆*sugE1*∆*sugE2* strains under low pH conditions. At pH 5, the ∆*sugE2* and ∆*sugE1*∆*sugE2* strains exhibited slightly better growth compared to WT and ∆*sugE1* (S5 Fig). At pH 3, ∆*sugE1*∆*sugE2* showed a more significant growth advantage (S5 Fig). Therefore, we suggest that the enhanced acid tolerance of the *sugE2*-deficient strain may contribute to its infection ability *in vivo*. Moreover, previous research has showed that the mutant of *acrB* downregulates the expression levels of SPI-1, SPI-2, and SPI-4, thereby reducing *S.* Typhimurium virulence [30]. Furthermore, inactivation of the efflux pump *acrD* gene leads to a decrease in the expression of SPI-1 in *S*. Typhimurium [57]. Mutations in TolC also reduce the secretion of the type III secretion system effectors SipA and SipC and diminish the invasion ability of HT-29 epithelial cells compared to the parent strain [58]. The higher expression level of SugE2 and the lower expression level of SPI-1 were observed in culture medium supplemented with intestinal extract, while the decreased transcription of SPI-1 genes was abolished in the Δ*sugE2* and Δ*sugE1*Δ*sugE2* strains (Figs 7 and 8). We propose that the efflux pump SugE2 senses intestinal signals to inhibit the expression level of the SPI-1 gene cluster, subsequently attenuating the virulence of *S*. 4,[5],12:i:- ZC055.

Both SugE1 and SugE2 are essential for *S*. 4,[5],12:i:- ZC055 resistance to DDAB, while only deletion of the *sugE2* result in the increase of *S*. 4,[5],12:i:- ZC055 virulence. The molecular docking simulations were performed to model the interaction between DDAB and SugE1/SugE2 proteins, predicting distinct binding sites for SugE1 and SugE2 proteins. Previous studies have identified the conserved amino acid residues in SugE proteins [59], and by aligning the amino acid sequences of SugE from different strains, we observed that SugE2 shares more conserved residues with *E. coli* and other strains. The molecular docking model revealed that Trp-3, Val-7, Ile-99, and Lys-102 are key residues involved in the binding of DDAB to SugE2. Among these, Trp-3, Ile-99, and Lys-102 are highly conserved across SugE protein from various strains, while Trp-22, Pro-23, and Phe-86 identified as critical binding sites for SugE1, show lower conservation (S7 Fig). These differences in substrate binding site may explain distinct roles of SugE1 and SugE2 involved in *S*. 4,[5],12:i:- ZC055 virulence.

**Fig 9:**
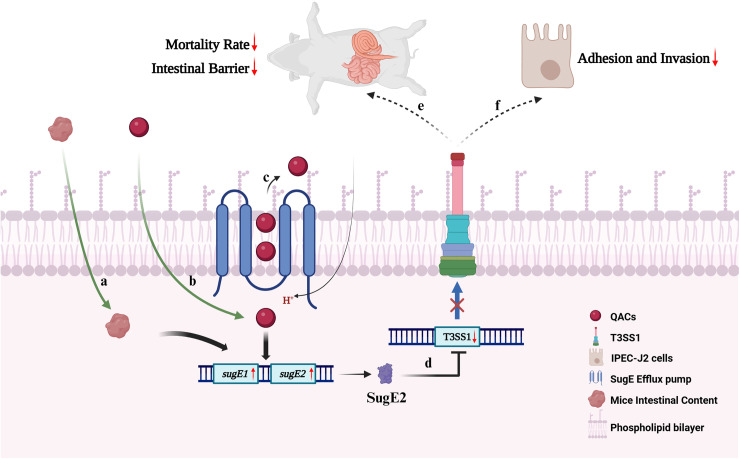
The contents of the mouse intestine (a) and quaternary ammonium compounds (b) can trigger the expression of the genes *sugE1* and *sugE2.* Subsequently, bacteria expel quaternary ammonium compounds via the SugE efflux pump **(c)**. Concurrently, the expression of the *sugE2* gene results in the downregulation of T3SS1 gene expression **(d)**, which leads to a reduced ability of the bacteria to adhere and invade to IPEC-J2 cells **(e)**, as well as their capacity to infect and colonize within the mouse host **(f)**. Created in BioRender. Wen, Y. (2025) https://BioRender.com/w33f839.

In summary, we propose that the following model for the role of the efflux pump SugE in resistance to DDAB disinfectant and bacterial virulence (Fig 9). We discovered the co-existence of *sugE1* and *sugE2* genes within the IncHI1B plasmid, which can efflux of QACs disinfectants, thereby conferring resistance to QACs in *S.* 4,[5],12:i:-. Moreover, our research demonstrated that SugE2 functions as an intestinal content sensor to downregulation of SPI-1 expression. This downregulation subsequently leads to the inhibition of adhesion, invasion, and virulence in *S*. 4,[5],12:i:-. Collectively, our findings unveiled a previously unnoticed role of the SMR-type efflux pump SugE2 in *Salmonella* virulence.

## Materials and methods

### Ethics statement

Animal experiments were performed according to the guide for the welfare and ethics of laboratory animals. All animals were housed in animal biosafety facilities in accordance with the procedures approved by the Institutional Animal Ethics Committee of Yangzhou University (reference number 202206004). All the animals were humanely handled.

### Bacterial stains, plasmids, and growth conditions

The strains and plasmids employed in this study were listed in S1 and S3 Tables. The primers employed in this study were shown in S4 Table. Bacteria were cultured in LB or M9 medium at 37°C, with antibiotics or supplements incorporated at appropriate concentrations. The deletion mutant and complemented strains were constructed as previously described [60].

### Susceptibility of *S*. 4,[5],12:i:- isolates to DDAB

The MICs for *S*. 4,[5],12:i:- in response to DDAB were determined following previously established protocols [61]. Briefly, DDAB was serially diluted two-fold in M9, MH broth or LB broth, with 100 µL of the dilution added to each well of a 96-well microtiter plate. Then, 100 µL of a suspension containing *S*. 4,[5],12:i:- was added to each well, and the plates were incubated at 37°C for 24 h. DDAB concentrations ranged from 1.5625 to 800 µg/mL. The MICs were determined as the lowest DDAB concentration capable of effectively inhibiting the visible growth of *S*. 4,[5],12:i:-. *S*. Typhimurium SL1344 and *E. coli* ATCC25922 were employed as control strains.

### SEM

The morphological changes in the ZC055 strain induced by treatment with DDAB were examined through SEM. The ZCO55 strain, cultured overnight in LB medium, was diluted to a concentration of 1 × 10^9^ CFU/mL and treated with varying concentrations of DDAB (0, 20, 100, 200, and 400 µg/mL) for 30 min. Subsequently, the bacteria were washed and fixed with 2.5% (w/v) glutaraldehyde at 4°C for 12 h. Following fixation, the bacteria underwent a triple wash in 0.1 M phosphate buffer and were subjected to dehydration in a graded series of ethanol concentrations (25%, 50%, 70%, 80%, and 90%) for 15 min each. The specimens were then examined using a GeminiSEM 300 (Carl Zeiss, Oberkochen, Germany).

### WGS

The genomic DNA of *S*. 4, [5], 12:i:- ZC055 was isolated using the TIANamp Bacteria DNA Kit (DP210831, Tiangen Biotech, China). This DNA was randomly fragmented into approximately 350-bp fragments employing a Covaris ultrasonic disruptor, followed by the construction of a sequencing library using the NEBNext DNA Library Prep Kit for Illumina (NEB, USA). Subsequently, the library was sequenced using the Illumina NovaSeq PE150 platform.

### RNA extraction, sequencing, and bioinformatics analyses

Three biological replicates of the ZC055 strain were cultured in LB broth, either supplemented with 100 µg/mL DDAB (1/2 MIC) or without DDAB, until reaching an optical density at 600 nm (OD_600_) of 0.6. Total RNA was extracted using the RNAprep Pure Cell/Bacteria Kit (DP430, Tiangen Biotech, China). The concentration of RNA was determined using the NanoDropTM One spectrophotometer (Thermo Fisher Scientific, USA), and DNA was carried out using DNase I (RNase-Free) (RT411, Tiangen Biotech, China). RNA-seq was conducted by Majorbio Co., Ltd. in China, utilizing the Illumina HiSeq 6000 system for paired-end sequencing. Bioinformatics analysis was performed on a cloud platform (Shanghai Majorbio Bio-Pharm Technology Co., Ltd., China). Reference genomes using WGS data from the ZC055 strain.

### qRT-PCR assays

Total RNA was treated with RNase-free DNase I to eliminate genomic DNA contamination. cDNA synthesis was conducted using reverse transcriptase (Vazyme, Nanjing, China). qRT-PCR was performed in triplicate, across three independent experiments, utilizing the universal SYBR green Master Mix (Vazyme, Nanjing, China) and specific primers (S3 Table). Transcript levels were normalized to that of *gyrB* for each sample using the ΔΔC_T_ method.

### Growth characteristic analysis

The *S*. 4,[5],12:i:- ZC055 strain, along with the Δ*sugE1*, Δ*sugE2*, and Δ*sugE1*Δ*sugE2* strains, were cultured in LB medium at 37°C for 12 h. Subsequently, these cultures were scaled up to 50 mL in LB and M9 media, with an initial OD_600_ of 0.05. OD_600_ values were measured hourly over a 24-h period to generate the growth curve.

### The survival of *Salmonella* treatment with CCCP and DDAB

*S*. 4,[5],12:i:- ZC055 strains were cultured at 37^o^C for 16 h with CCCP (MCE, USA) at final concentrations of 0, 30, and 60 µM. After incubation, the bacterial suspensions were collected and the OD_600_ was adjusted to 0.1. The bacterial suspensions were treated with two concentrations of DDAB (100 μg/mL and 50 μg/mL) and incubated at 37^o^C. At specific time points (0, 5, 10, 20, and 30 minutes), 100 μL aliquots were withdrawn and subjected to serial dilution. The diluted samples were plated on the LB agar plates and incubated overnight at 37^o^C for colony counting.

### H33342 accumulation and ethidium bromide efflux assay

H33343 accumulation and ethidium bromide efflux was assessed as previously described [33]. Cultures with an OD_600_ of 0.6 were dispensed into a 96-well black plate, and fluorescence measurements were acquired at 10-min intervals over a 100-min period using a Spark microplate reader. Initial fluorescence readings were taken 5 min prior to the addition of H33342 (2.5 μM) or glucose (25 mM), respectively. For the examination of ethidium bromide efflux, cultures were pre-treated with ethidium bromide (50 μg/mL) and CCCP at a concentration of 100 μM.

### Interaction models of DDAB with the SugE1 or SugE2 protein

Homology modeling was performed using the 3D structure of the protein PDB ID 6wk5 as a template, aligning it with the amino acid sequences of SugE1 and SugE2. The resulting GMQE values were 0.73 and 0.74, respectively (GMQE values exceeding 0.5, indicating structural reliability. Subsequently, PyMOL 2.3.0 software was utilized to examine the protein structures. For molecular docking studies, DDAB was employed as the small molecule ligand, and its 3D structure file was retrieved from the PubChem database. Molecular docking was executed using AutoDock Vina 1.2.0.

### Adhesion and invasion assays

IPEC-J2 cells were transferred to 24-well plates and cultured at 37°C in a 5% CO_2_ environment. *S*. 4,[5],12:i:- WT, Δ*sugE1*, Δ*sugE2*, and Δ*sugE1*Δ*sugE2* strains, previously cultured overnight, were diluted into fresh LB medium, with or without varying concentrations of DDAB. The IPEC-J2 cells were then infected with the *S*. 4,[5],12:i:- strains at a MOI of 100:1. For adhesion assays, the IPEC-J2 cells were rinsed twice with PBS after a 2-h infection period. Subsequently, they were incubated in PBS containing 0.2% Triton X-100 at 37°C for 15 min. The lysate was diluted, and the bacterial count was determined using LB agar. For invasion assays, following a 2-h infection period, the IPEC-J2 cells were treated with 100 μg/mL gentamicin for 1 h and then cultured for an additional 2 h. The bacterial count was determined using LB agar.

### Mouse experiments

Six to eight-week-old C57BL/6 mice were divided into five groups (n = 7) to assess the virulence of *sugE1* or/and *sugE2* mutant strains. The mice underwent a 4-h period of fasting and water deprivation, followed by oral gavage administration of 7.5 mg of streptomycin, which continued for 20 h. Each group was orally infected with 3 × 10^7^ CFUs of ZC055, Δ*sugE1*, Δ*sugE2*, or Δ*sugE1*Δ*sugE2*, while the control group was administered 100 μL of sterile PBS. Survival and body weight were continuously monitored for 18 days. In addition, we evaluated the ability of *sugE1* or/and *sugE2* mutant strains to colonize in mice. Six to eight-week-old C57BL/6 mice were divided into five groups (n = 5). The mice underwent a 10-h period of fasting and water deprivation, followed by oral gavage administration 100 μL of 2% NaHCO_3_, which continued for 2 h. Each group was orally infected with 3 × 10^5^ CFUs of ZC055, Δ*sugE1*, Δ*sugE2*, or Δ*sugE1*Δ*sugE2*, while the control group was administered 100 μL of sterile PBS. At three dpi, the liver, spleen, and cecum were collected to determine the live bacterial count. Fresh intestinal tissue was promptly placed in a fixation solution for a minimum of 24 h. Paraffin-embedded slides were prepared and stained with H&E, and basic pathological changes as well as typical lesion sites were observed under a microscope.

### Statistical analyses

All experiments were conducted a minimum of three times. The statistical significance of survival curves was assessed through a two-tailed log-rank (Mantel-Cox) test. Mouse experiments employed a two-tailed Mann-Whitney U test, whereas other experiments utilized a Student’s *t*-test. A *p* value of < 0.05 was considered statistically significant and is denoted as “significant” in the text. In graphical representations of the data, *** marks *p* value < 0.001; ** is *p* value < 0.01, * is *p* value < 0.05.

## Supporting information

S1 Fig Comparative growth dynamics of the *S.* 4,[5],12:i:- ZC055, *ΔsugE1*, *ΔsugE2*, and Δ*sugE1*Δ*sugE2 strains .
* (A) the growth curve and generation times of the strains in LB broth. (B) the same parameters in M9 minimal medium. The depicted growth curves are the mean values, derived from three independent.
(S1FIG)

S2 Fig Growth curve of ZC055 in response to various concentrations of CCCP at 0, 30, and 60 μM. (TIF)

S3 Fig Interaction of DDAB with SugE1 (A) or SugE2 (B) protein. The visualization illustrates DDAB (white) and the amino acid residues (blue) in the proteins that interact with the DDAB. Hydrocarbon bonding interactions are represented by pale green dashed lines, while Alkyl/π-Alkyl interactions are indicated by pale pink lines.
(TIF)

S4 Fig The ZC055, Δ *sugE1*, Δ *sugE2*, and Δ *sugE1* Δ *sugE2* strains were intraperitoneally injected into C57BL/6 mice. Kaplan–Meier survival curves of mice for 18 days after infection. The *P* value was determined using a log rank (Mantel–Cox) test. The Y-axis represents the percentage of survival, while the X-axis represents time post-injection in days.
(TIF)

S5 Fig Comparative growth dynamics of *S .
* **4,[5],12:i:- ZC055, Δ*sugE1*, Δ*sugE2* and Δ*sugE1*Δ*sugE2* strains in various pH conditions.** The growth of both strains was monitored under different pH environments, including pH 3.0 (A), pH 5.0 (B), and pH 7.0 (C) in LB broth. The displayed growth curves are representative of three independent experiments, indicating the mean values.
(TIF)

S6 Fig (A) The motility of ZC055, Δ*sugE1*, Δ*sugE2* and Δ*sugE1*Δ*sugE2* was evaluated in LB agar medium. (B) The cytotoxicity of the Δ*sugE1*, Δ*sugE2*, and Δ*sugE1*Δ*sugE2* strains in IPEC-J2 cells was assessed and compared to the cytotoxicity of the ZC055 strain in IPEC-J2 cells.
(TIF)

S7 Fig The alignments of SugE proteins across different bacterial species. The red box indicates the conserved amino acids binding sites in the SugE2 protein.
(TIF)

S1 Table Metadata, including source, year of isolation and Origin of the *S.* 4,[5],12:i:- isolates included in this study. (DOCX)

S2 Table Summary of antibiotic resistance and virulence genes in IncHI1B plasmid. (DOCX)

S3 Table Bacterial strains and plasmids used in this study. (DOCX)

S4 Table Primers used in this study. (DOCX)
