# Supplementary figures and images for: The efflux pump SugE2 involved in protection of Salmonella 4,[5],12:i:- against quaternary ammonium salts and inhibition of virulence
Source: PLoS Pathog. 2025 Mar 18;21(3):e1012951. doi: 10.1371/journal.ppat.1012951 (PMC11918376; doi:10.1371/journal.ppat.1012951)

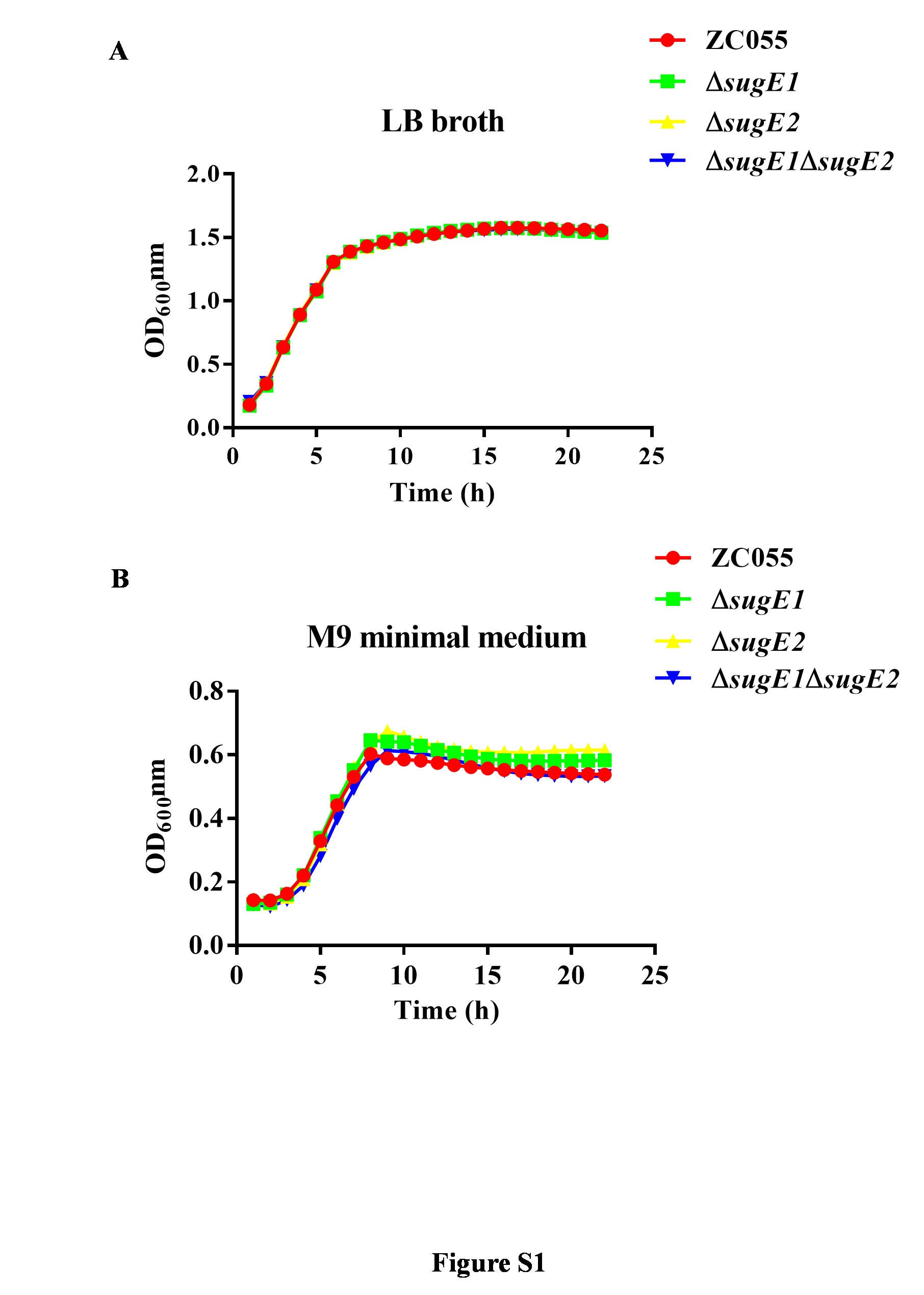

Supplement: S1 Fig — (A) the growth curve and generation times of the strains in LB broth. (B) the same parameters in M9 minimal medium. The depicted growth curves are the mean values, derived from three independent. (S1FIG) [file ppat.1012951.s001.tif]

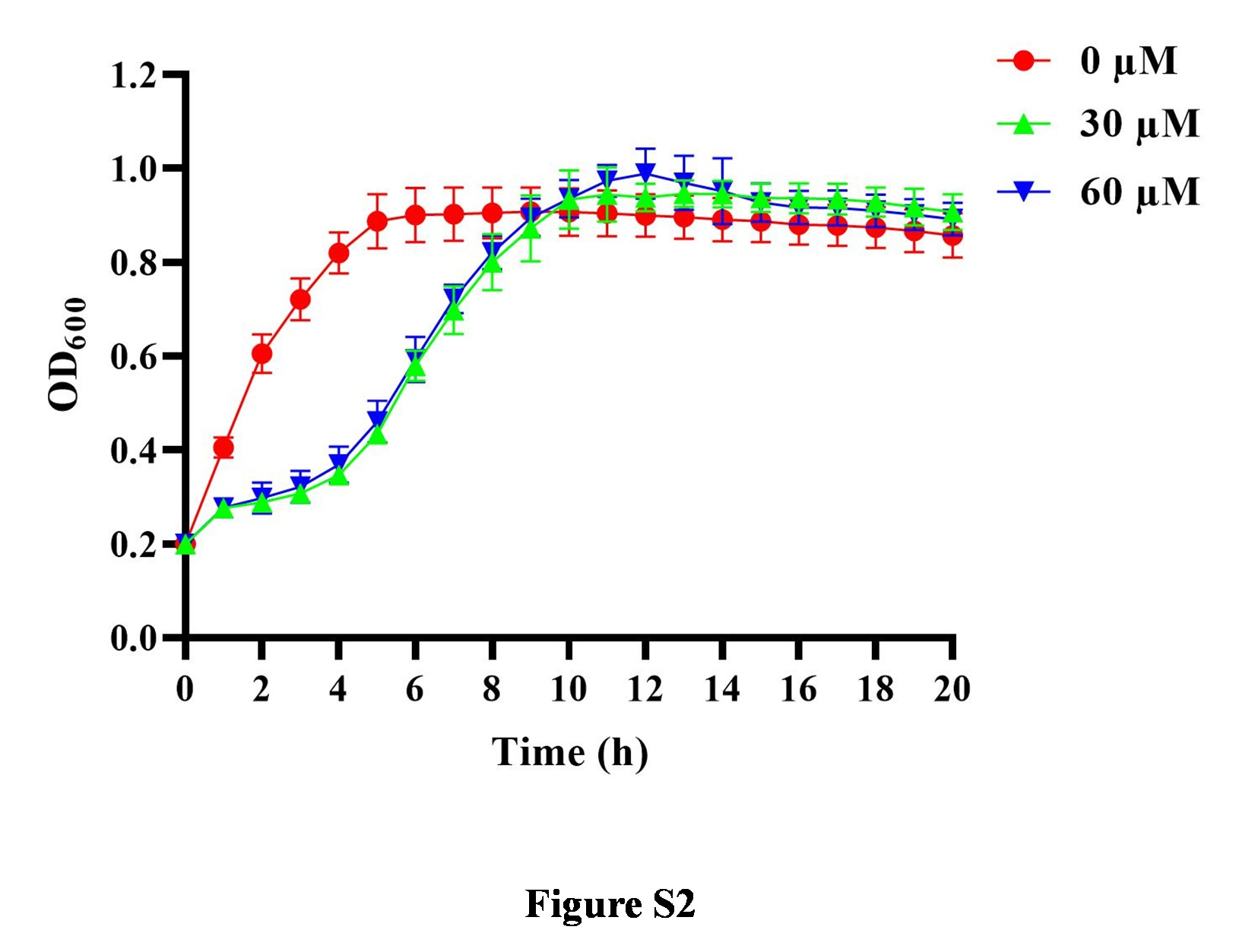

Supplement: S2 Fig — (TIF) [file ppat.1012951.s002.tif]

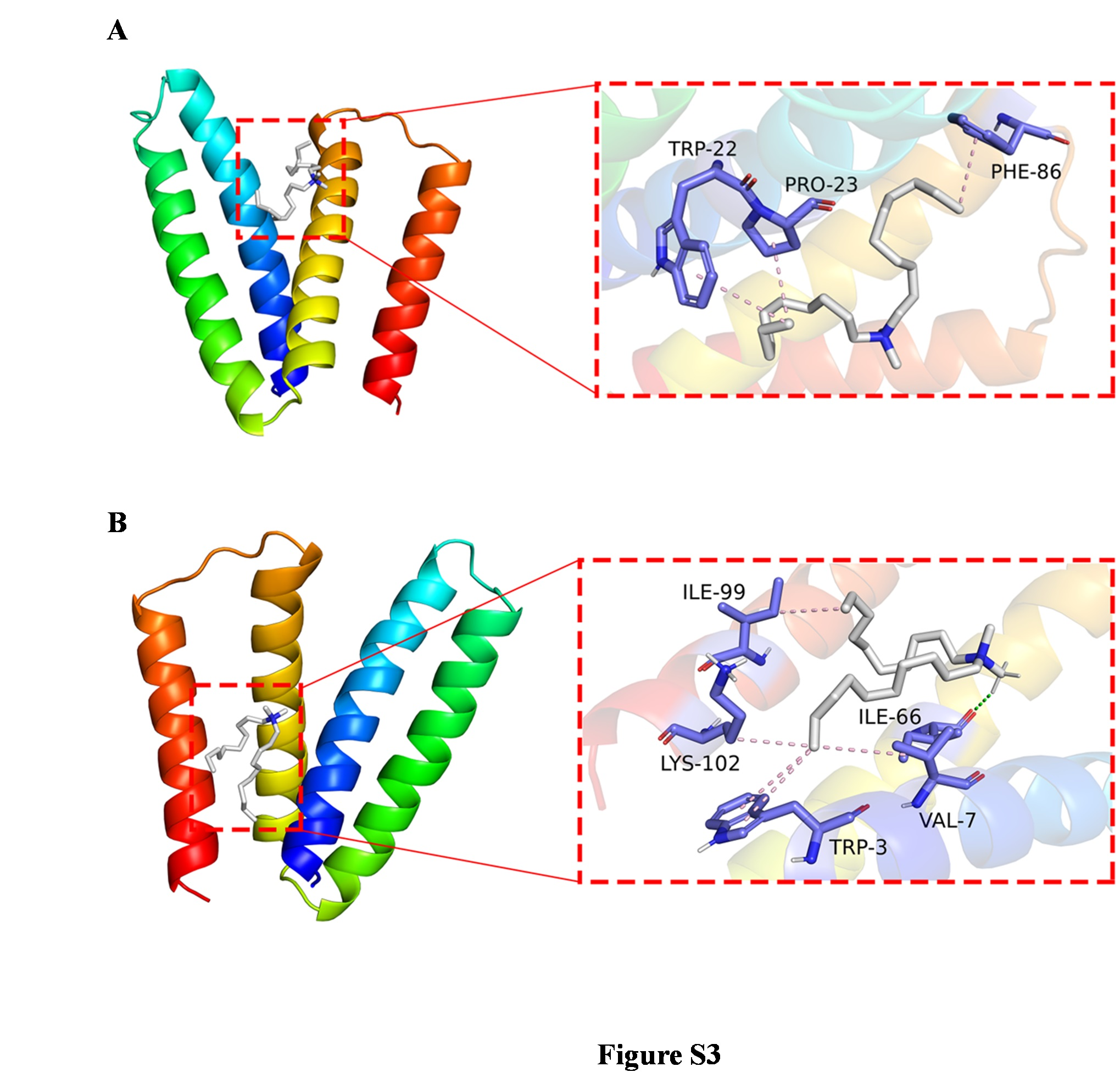

Supplement: S3 Fig — The visualization illustrates DDAB (white) and the amino acid residues (blue) in the proteins that interact with the DDAB. Hydrocarbon bonding interactions are represented by pale green dashed lines, while Alkyl/π-Alkyl interactions are indicated by pale pink lines. (TIF) [file ppat.1012951.s003.tif]

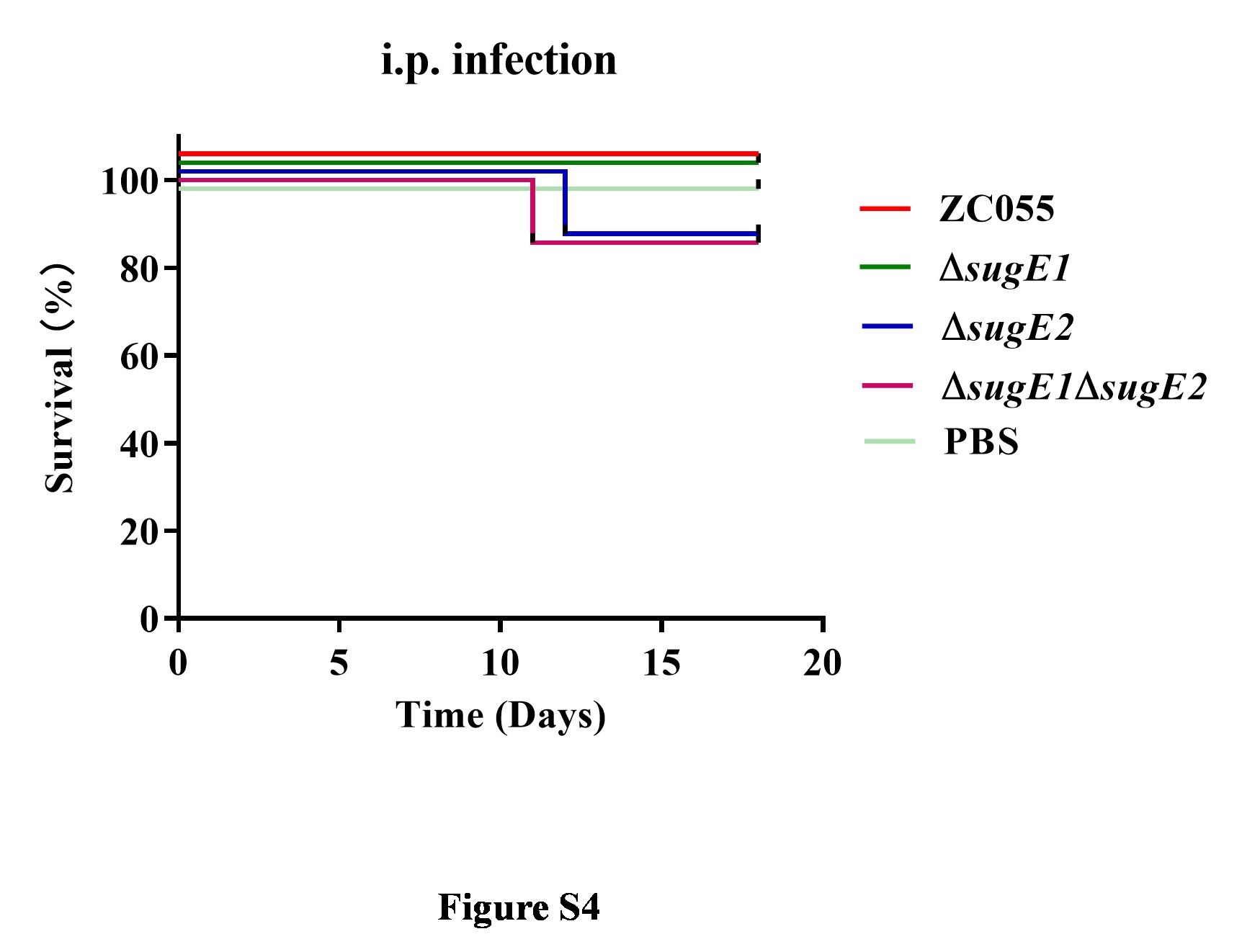

Supplement: S4 Fig — Kaplan–Meier survival curves of mice for 18 days after infection. The P value was determined using a log rank (Mantel–Cox) test. The Y-axis represents the percentage of survival, while the X-axis represents time post-injection in days. (TIF) [file ppat.1012951.s004.tif]

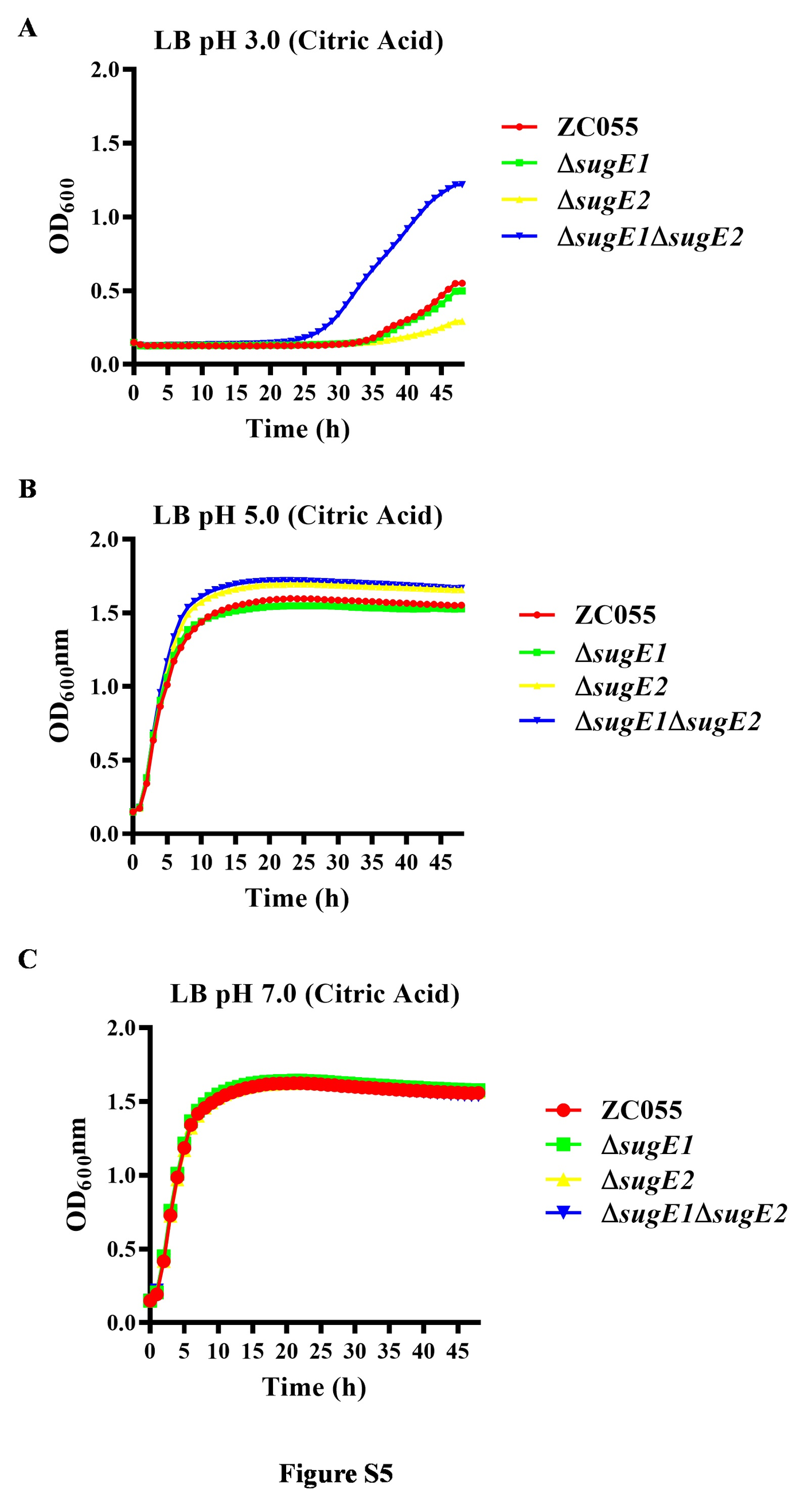

Supplement: S5 Fig — 4,[5],12:i:- ZC055, ΔsugE1, ΔsugE2 and ΔsugE1ΔsugE2 strains in various pH conditions. The growth of both strains was monitored under different pH environments, including pH 3.0 (A), pH 5.0 (B), and pH 7.0 (C) in LB broth. The displayed growth curves are representative of three independent experiments, indicating the mean values. (TIF) [file ppat.1012951.s005.tif]

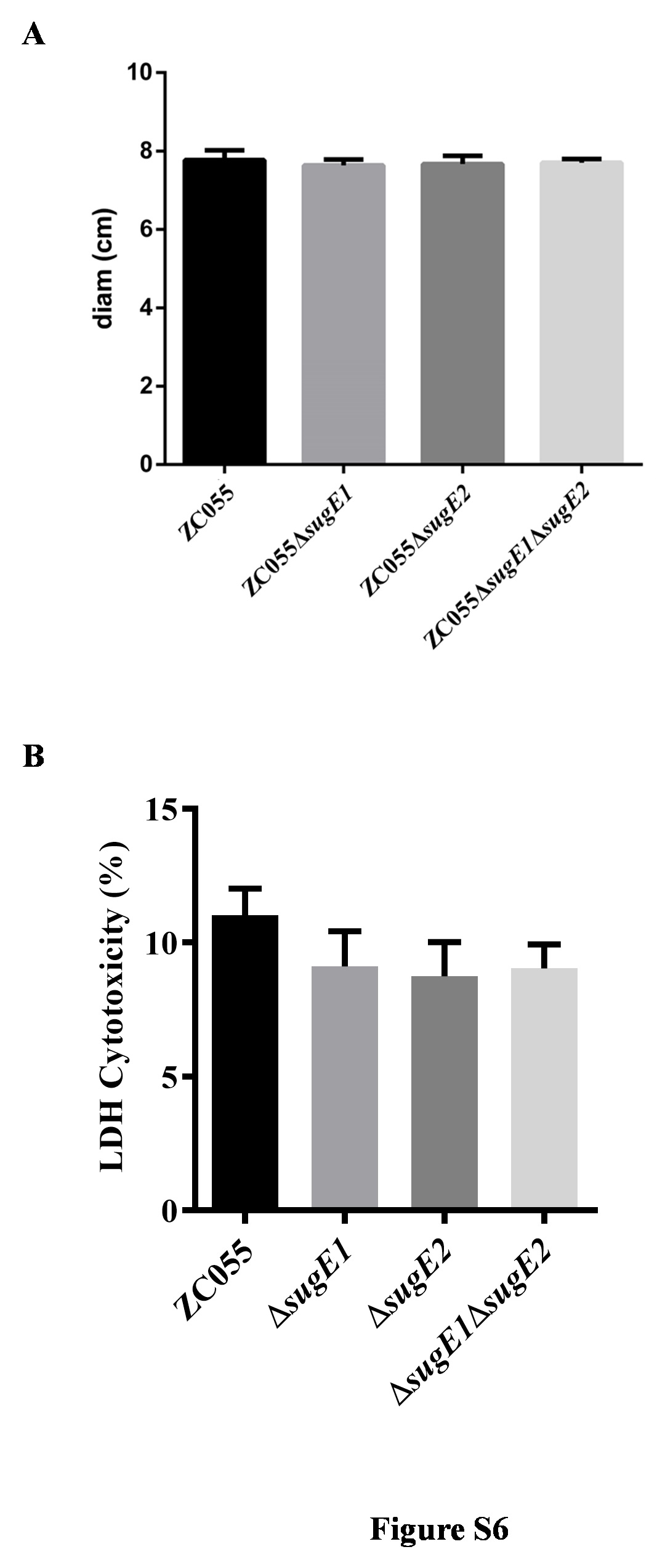

Supplement: S6 Fig — (B) The cytotoxicity of the ΔsugE1, ΔsugE2, and ΔsugE1ΔsugE2 strains in IPEC-J2 cells was assessed and compared to the cytotoxicity of the ZC055 strain in IPEC-J2 cells. (TIF) [file ppat.1012951.s006.tif]

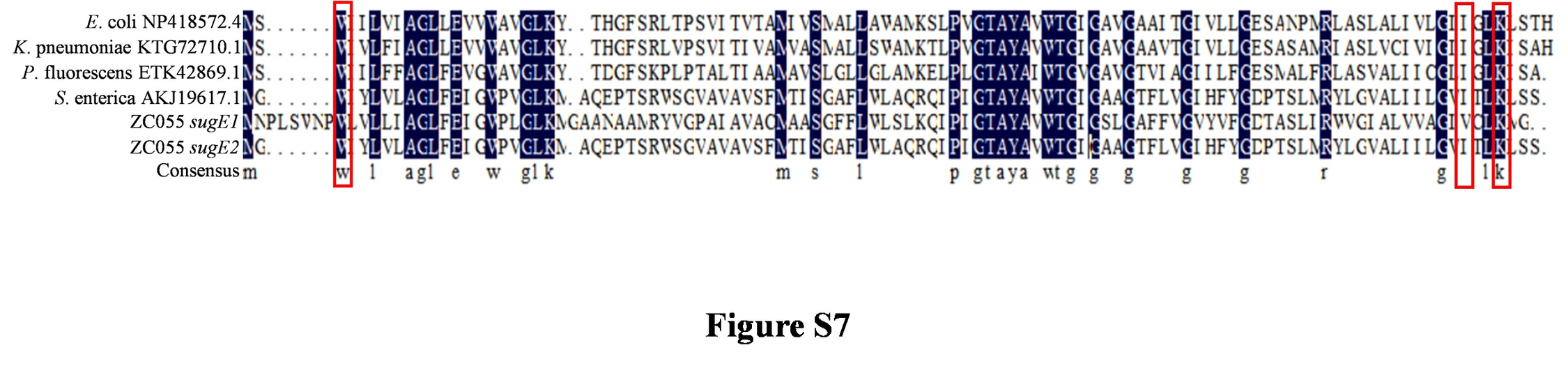

Supplement: S7 Fig — The red box indicates the conserved amino acids binding sites in the SugE2 protein. (TIF) [file ppat.1012951.s007.tif]
