# Supplementary material for: The efflux pump SugE2 involved in protection of Salmonella 4,[5],12:i:- against quaternary ammonium salts and inhibition of virulence
Source: PLoS Pathog. 2025 Mar 18;21(3):e1012951. doi: 10.1371/journal.ppat.1012951 (PMC11918376; doi:10.1371/journal.ppat.1012951)
Supplement: S1 Table — (DOCX) [file ppat.1012951.s008.docx]

**S1 Table. Metadata, including source, year of isolation and Origin of the *S.* 4,[5],12:i:- isolates included in this study**

| Isolate ID | Source | Sampling stage/Category | Year | M9 medium | MH broth |
| --- | --- | --- | --- | --- | --- |
|  |  |  |  | MIC（μg/mL） | |
| SH032 | Nursery - Porcine faeces | Faecal sample | 2016 | 50 | 100 |
| SH034 | Finish House - Porcine faeces | Faecal sample | 2016 | 12.5 | 25 |
| SH035 | Gilt - feed | Feed sample | 2016 | 12.5 | 25 |
| SH037 | Piglet - feed | Feed sample | 2016 | 12.5 | 25 |
| SH044 | Nursery - Porcine faeces | Faecal sample | 2016 | 12.5 | 25 |
| SH046 | Nursery - Porcine faeces | Faecal sample | 2016 | 12.5 | 25 |
| SH058 | Finish House - Porcine faeces | Faecal sample | 2016 | 12.5 | 25 |
| SH059 | Finish House - Floor swab | Environmental swab | 2016 | 12.5 | 25 |
| SH060 | Finish House - Water Nozzle swab | Environmental swab | 2016 | 12.5 | 25 |
| SH062 | Finish House - Corral swab | Environmental swab | 2016 | 12.5 | 25 |
| SH063 | Farrowing House - Floor swab | Environmental swab | 2016 | 12.5 | 25 |
| SH065 | Finish House - Trough swab | Environmental swab | 2016 | 12.5 | 25 |
| SH066 | Farrowing House - Floor swab | Environmental swab | 2016 | 12.5 | 25 |
| SH068 | Nursery - Trough swab | Environmental swab | 2016 | 12.5 | 25 |
| SH069 | Nursery - Floor swab | Environmental swab | 2016 | 12.5 | 25 |
| SH070 | Nursery - Floor swab | Environmental swab | 2016 | 12.5 | 25 |
| SH071 | Nursery - Water Nozzle swab | Environmental swab | 2016 | 12.5 | 25 |
| SH073 | Finish House - Stockman's Hand swab | Environmental swab | 2016 | 12.5 | 25 |
| SH074 | Finish House - Corral swab | Environmental swab | 2016 | 12.5 | 25 |
| SH076 | Finish House - Floor swab | Environmental swab | 2016 | 12.5 | 25 |
| SH077 | Finish House - Floor swab | Environmental swab | 2016 | 12.5 | 25 |
| SH079 | Gilt - Porcine faeces | Faecal sample | 2017 | 6.25 | 25 |
| SH080 | Gilt - Porcine faeces | Faecal sample | 2017 | 12.5 | 25 |
| SH081 | Finish House - Porcine faeces | Faecal sample | 2017 | 12.5 | 25 |
| SH082 | Finish House - Porcine faeces | Faecal sample | 2017 | 12.5 | 25 |
| SH084 | Mating House - Floor swab | Environmental swab | 2017 | 12.5 | 25 |
| SH085 | Mating House - Floor swab | Environmental swab | 2017 | 12.5 | 25 |
| SH087 | Nursery - Porcine faeces | Faecal sample | 2017 | 12.5 | 25 |
| SH088 | Nursery - Porcine faeces | Faecal sample | 2017 | 12.5 | 25 |
| SH089 | Finish House - Porcine faeces | Faecal sample | 2017 | 12.5 | 25 |
| SH092 | Finish House - Porcine faeces | Faecal sample | 2017 | 12.5 | 25 |
| SH093 | Finish House - Porcine faeces | Faecal sample | 2017 | 12.5 | 25 |
| SH094 | Finish House - Porcine faeces | Faecal sample | 2017 | 12.5 | 25 |
| SH096 | Finish House - Porcine faeces | Faecal sample | 2017 | 12.5 | 25 |
| SH098 | Finish House - Porcine faeces | Faecal sample | 2017 | 12.5 | 25 |
| SH099 | Finish House - Porcine faeces | Faecal sample | 2017 | 6.25 | 25 |
| SH100 | Finish House - Porcine faeces | Faecal sample | 2017 | 12.5 | 25 |
| SH101 | Finish House - Porcine faeces | Faecal sample | 2017 | 12.5 | 25 |
| SH102 | Finish House - Porcine faeces | Faecal sample | 2017 | 12.5 | 25 |
| SH104 | Nursery - Porcine faeces | Faecal sample | 2017 | 12.5 | 25 |
| SH105 | Nursery - Porcine faeces | Faecal sample | 2017 | 12.5 | 25 |
| SH106 | Finish House - Porcine faeces | Faecal sample | 2017 | 12.5 | 25 |
| SH108 | Finish House - Porcine faeces | Faecal sample | 2017 | 12.5 | 25 |
| SH109 | Finish House - Porcine faeces | Faecal sample | 2017 | 12.5 | 25 |
| SH110 | Finish House - Porcine faeces | Faecal sample | 2017 | 12.5 | 25 |
| SH111 | Finish House - Porcine faeces | Faecal sample | 2017 | 12.5 | 25 |
| SH116 | Farrowing House - Porcine faeces | Faecal sample | 2017 | 12.5 | 25 |
| SH120 | Finish House - Porcine faeces | Faecal sample | 2017 | 12.5 | 25 |
| SH122 | Finish House - Porcine faeces | Faecal sample | 2017 | 6.25 | 25 |
| SH123 | Finish House - Porcine faeces | Faecal sample | 2017 | 12.5 | 25 |
| SH124 | Finish House - Porcine faeces | Faecal sample | 2017 | 12.5 | 25 |
| SH134 | Mating House - Weaned Sow faeces | Faecal sample | 2017 | 12.5 | 25 |
| SH136 | Mating House - Weaned Sow faeces | Faecal sample | 2017 | 12.5 | 25 |
| SH141 | Farrowing House - Porcine faeces | Faecal sample | 2017 | 12.5 | 25 |
| SH149 | Finish House - Porcine faeces | Faecal sample | 2017 | 12.5 | 25 |
| SH164 | Newly Purchased Pig - Porcine faeces | Faecal sample | 2017 | 12.5 | 25 |
| SH165 | Newly Purchased Pig - Porcine faeces | Faecal sample | 2017 | 12.5 | 25 |
| SH169 | Nursery - Porcine faeces | Faecal sample | 2017 | 12.5 | 25 |
| SH170 | Nursery - Porcine faeces | Faecal sample | 2017 | 12.5 | 25 |
| SH171 | Finish House - Porcine faeces | Faecal sample | 2017 | 12.5 | 25 |
| SH172 | Finish House - Porcine faeces | Faecal sample | 2017 | 12.5 | 25 |
| SH173 | Finish House - Porcine faeces | Faecal sample | 2017 | 50 | 50 |
| SH174 | Finish House - Porcine faeces | Faecal sample | 2017 | 12.5 | 25 |
| SH175 | Finish House - Porcine faeces | Faecal sample | 2017 | 12.5 | 25 |
| SH176 | Finish House - Porcine faeces | Faecal sample | 2017 | 12.5 | 25 |
| SH177 | Finish House - Porcine faeces | Faecal sample | 2017 | 12.5 | 25 |
| SH189 | Finish House - Porcine faeces | Faecal sample | 2018 | 12.5 | 25 |
| SH192 | Finish House - Porcine faeces | Faecal sample | 2018 | 12.5 | 25 |
| SH193 | Finish House - Porcine faeces | Faecal sample | 2018 | 12.5 | 25 |
| ZC004 | Nursery - Porcine faeces | Faecal sample | 2018 | 50 | 200 |
| ZC006 | Nursery - Porcine faeces | Faecal sample | 2018 | 12.5 | 25 |
| ZC007 | Nursery - Porcine faeces | Faecal sample | 2018 | 12.5 | 25 |
| ZC008 | Nursery - Porcine faeces | Faecal sample | 2018 | 12.5 | 25 |
| ZC009 | Nursery - Porcine faeces | Faecal sample | 2018 | 12.5 | 25 |
| ZC010 | Nursery - Porcine faeces | Faecal sample | 2018 | 12.5 | 25 |
| ZC011 | Finish House - Porcine faeces | Faecal sample | 2018 | 6.25 | 25 |
| ZC013 | Finish House - Porcine faeces | Faecal sample | 2018 | 12.5 | 25 |
| ZC016 | Finish House - Porcine faeces | Faecal sample | 2018 | 12.5 | 25 |
| ZC026 | Boar - feed | Feed sample | 2018 | 12.5 | 25 |
| ZC027 | Farrowing House - Porcine faeces | Faecal sample | 2018 | 12.5 | 25 |
| ZC029 | Farrowing House - Porcine faeces | Faecal sample | 2018 | 12.5 | 25 |
| ZC030 | Farrowing House - Porcine faeces | Faecal sample | 2018 | 12.5 | 25 |
| ZC038 | Finish House - Porcine faeces | Faecal sample | 2018 | 12.5 | 25 |
| ZC040 | Finish House - Porcine faeces | Faecal sample | 2018 | 12.5 | 25 |
| ZC041 | Finish House - Porcine faeces | Faecal sample | 2018 | 12.5 | 25 |
| ZC042 | Finish House - Porcine faeces | Faecal sample | 2018 | 12.5 | 25 |
| ZC043 | Finish House - Porcine faeces | Faecal sample | 2018 | 12.5 | 25 |
| ZC048 | Finish House - Porcine faeces | Faecal sample | 2018 | 12.5 | 25 |
| ZC050 | Farrowing House - Porcine faeces | Faecal sample | 2018 | 100 | 200 |
| ZC055 | Weaned Sow - Porcine faeces | Faecal sample | 2018 | 100 | 200 |
| ZC057 | Nursery - Porcine faeces | Faecal sample | 2018 | 6.25 | 25 |
| ZC058 | Nursery - Porcine faeces | Faecal sample | 2018 | 12.5 | 25 |
| ZC071 | Mating House - Porcine faeces | Faecal sample | 2018 | 12.5 | 25 |
| ZC095 | Finish House - Porcine faeces | Faecal sample | 2018 | 12.5 | 25 |
| ZC098 | Fattening Pig - feed | Feed sample | 2018 | 12.5 | 25 |
| ZC102 | Nursery - Porcine faeces | Faecal sample | 2018 | 12.5 | 25 |
| ZC115 | Finish House - Porcine faeces | Faecal sample | 2018 | 12.5 | 25 |
| ZC117 | Finish House - Porcine faeces | Faecal sample | 2018 | 12.5 | 25 |
| ZC122 | Finish House - Porcine faeces | Faecal sample | 2018 | 12.5 | 25 |
| ZC124 | Finish House - Porcine faeces | Faecal sample | 2018 | 12.5 | 25 |
| ZC129 | Nursery - Porcine faeces | Faecal sample | 2018 | 12.5 | 25 |
| ZC130 | Nursery - Porcine faeces | Faecal sample | 2018 | 12.5 | 25 |
| ZC161 | Finish House - Porcine faeces | Faecal sample | 2018 | 12.5 | 25 |
| ZC163 | Finish House - Porcine faeces | Faecal sample | 2018 | 12.5 | 25 |
| ZC201 | Finish House - Porcine faeces | Faecal sample | 2018 | 100 | 200 |
| ZC205 | Finish House - Porcine faeces | Faecal sample | 2018 | 12.5 | 25 |
| ZC208 | Finish House - Porcine faeces | Faecal sample | 2018 | 12.5 | 25 |
| ZC223 | Finish House - Porcine faeces | Faecal sample | 2019 | 12.5 | 25 |
| ZC230 | Nursery - Porcine faeces | Faecal sample | 2019 | 12.5 | 25 |
| FB10 | Laboratory | —— | —— | 12.5 | 25 |
| 23P1 | Laboratory | —— | —— | 6.25 | 12.5 |
| 2457 | Laboratory | —— | —— | 12.5 | 12.5 |
| ATCC25922 | Laboratory | —— | —— | 6.25 | 12.5 |
| SL1344 | Laboratory | —— | —— | 3.125 | 6.25 |
