# Supplementary material for: The efflux pump SugE2 involved in protection of Salmonella 4,[5],12:i:- against quaternary ammonium salts and inhibition of virulence
Source: PLoS Pathog. 2025 Mar 18;21(3):e1012951. doi: 10.1371/journal.ppat.1012951 (PMC11918376; doi:10.1371/journal.ppat.1012951)
Supplement: S2 Table — (DOCX) [file ppat.1012951.s009.docx]

**S2 Table. Summary of antibiotic resistance and virulence genes in IncHI1B plasmid**

| Category | Gene Name | Gene Function |
| --- | --- | --- |
| Virulence Genes |  |  |
|  | *virB* | Lvh (Legionella vir homologs) type IVA secretion system, *virB* LvhB11 |
|  | *adhD* | *adhD* Putative zinc-type alcohol dehydrogenase AdhD aldehyde reductase, MymA operon |
|  | *pilW* | *pilW* putative transposase, Type IV pili |
| Resistance Genes |  |  |
|  | *aac(6')-Ib-cr* | aminoglycoside resistance protein, antibiotic inactivation enzyme, fluoroquinolone resistance protein |
|  | *bla*_OXA-1_ | antibiotic inactivation enzyme, beta-lactam resistance protein |
|  | *catB3* | antibiotic inactivation enzyme, phenicol resistance protein |
|  | *arr-3* | antibiotic inactivation enzyme, rifamycin resistance protein |
|  | *sul1* | antibiotic target replacement protein, sulfonamide resistance protein |
