# Supplementary material for: The efflux pump SugE2 involved in protection of Salmonella 4,[5],12:i:- against quaternary ammonium salts and inhibition of virulence
Source: PLoS Pathog. 2025 Mar 18;21(3):e1012951. doi: 10.1371/journal.ppat.1012951 (PMC11918376; doi:10.1371/journal.ppat.1012951)
Supplement: S3 Table — (DOCX) [file ppat.1012951.s010.docx]

**S3 Table. Bacterial strains and plasmids used in this study**

| Strain or plasmid | Relevant characteristics | Reference |
| --- | --- | --- |
| ***Escherichia coli*** |  |  |
| X7213 λ*pir* | Host for π requiring plasmids, conjugal donor | Labortaory collection |
| DH5α λ*pir* | Host for π requiring plasmids | Labortaory collection |
| C600 | Recipient bacterial strain for plasmid conjugation | Labortaory collection |
| ATCC25922 | MIC quality control bacterial strain | Labortaory collection |
| ***Salmonella*** |  |  |
| ZC055 | Wild type | This study |
| Δ*sugE1* | ZC055, In-frame deletion in *sugE1* | This study |
| Δ*sugE2* | ZC055, In-frame deletion in *sugE2* | This study |
| Δ*sugE1*Δ*sugE2* | ZC055, In-frame deletion in *sugE1* and *sugE2* | This study |
| Δ*sugE1*::*sugE1* | Δ*sugE1* with pMMB207 expressing the *sugE1* gene, Cm^r^ | This study |
| Δ*sugE2*::*sugE2* | Δ*sugE2* with pMMB207 expressing the *sugE2* gene, Cm^r^ | This study |
| FFLB | Wild type | This study |
| **Plasmids** |  |  |
| pDM4 | Suicide vector, pir dependent, R6K, SacBR, Cm^r^ | (1) |
| pMMB207 | oriR(RSF1010), lacIq Cmrp tac oriT MCS | (2) |

**REFFERENCE**

1. Wang S, Lauritz J, Jass J, Milton DL: A ToxR homolog from *Vibrio anguillarum* serotype O1 regulated its own production, bile resistance, and biofilm formation. J Bacteriol. 2002, 184: 1630-1639. 10.1128/JB.184.6.1630-1639.2002.

2. Morales VM, Bäckman A, Bagdasarian M. A series of wide-host-range low-copy-number vectors that allow direct screening for recombinants. Gene. 1991;97:39–47.
