# Supplementary material for: The efflux pump SugE2 involved in protection of Salmonella 4,[5],12:i:- against quaternary ammonium salts and inhibition of virulence
Source: PLoS Pathog. 2025 Mar 18;21(3):e1012951. doi: 10.1371/journal.ppat.1012951 (PMC11918376; doi:10.1371/journal.ppat.1012951)
Supplement: S4 Table — (DOCX) [file ppat.1012951.s011.docx]

**S4 Table. Primers used in this study**

| Primer name | Primer sequence (5' to 3') |
| --- | --- |
| *sugE1*-F | ATGAACCCGTTGTCATGGAATCCAT |
| *sugE1*-R | TTATCCCATTTTCAGACAGACGATG |
| *sugE2*-F | TCAACTCGATAACTTGAGCGTGATA |
| *sugE2*-R | ATGGGCTGGATCTATCTTGTTTTGG |
| IncHI1B-F | CTGTTGCCACCGTCTTTCCTGAG |
| IncHI1B-R | GGATGCTCTTTACGGATTTGTCCC |
| pDM4-F | GGTGCTCCAGTGGCTTCTGTTTCTA |
| pDM4-R | CAGCAACTTAAATAGCCTCTAAGGT |
| *sugE1*-up-F | GAGCGGATAACAATTTGTGGAATCCCGGGAGGCAACAGGCACTCCTTTCA |
| *sugE1*-up-R | ACAGCCAGATAATAGGCAGTAGGCGGTCAA |
| *sugE1*-down-F | ACTGCCTATTATCTGGCTGTGCTTCAAACG |
| *sugE1*-down-R | AGCGGAGTGTATATCAAGCTTATCGATACCTTCATCTACTGCCTCAAACTCC |
| *sugE2*-up-F | GAGCGGATAACAATTTGTGGAATCCCGGGACTCGAAGGCTCGCTGAAGATC |
| *sugE2*-up-R | ACGCTGGAGTCGGTCACCTCCTCCTAGTCGTTAT |
| *sugE2*-down-F | GAGGTGACCGACTCCAGCGTGACCACCAGC |
| *sugE2*-down-R | AGCGGAGTGTATATCAAGCTTATCGATACCGCCAGCGAGCCGTCAGAAAT |
| *sugE1*-in-F | AGATGGGTGCGGCCAATGCA |
| *sugE1*-in-R | AACGTATCAAGGAGGCGGTAT |
| *sugE1*-out-F | ATGCCATAGAGCTTGGTGGA |
| *sugE1*-out-R | TCGGAAAGTGTCGGGTTGAT |
| *sugE2*-in-F | CGCATCAGGGAGGTTGGGTC |
| *sugE2*-in-R | CAACATCGCGTTGGAGTGGG |
| *sugE2*-out-F | TCACGCAGGAAACGCAGAAG |
| *sugE2*-out-R | CCCTTGCTATCCCACAAACAGA |
| pMMB207-F | GAGCGGATAACAATTTCACACAGG |
| pMMB207-R | GATTTAATCTGTATCAGG |
| pMMB207-*sugE1*-F | TCGGTACCCGGGGATCCTCTAGTAAGGAGGTAGGATAATAATGAACCCGTTGTCATGGAATCCAT |
| pMMB207-*sugE1*-R | TCCGCCAAAACAGCCAAGCTTTATTATCCCATTTTCAGACAGACGATG |
| pMMB207-*sugE2*-F | TCGGTACCCGGGGATCCTCTAGTAAGGAGGTAGGATAATATCAACTCGATAACTTGAGCGTGATA |
| pMMB207-*sugE2*-R | TCCGCCAAAACAGCCAAGCTTTAATGGGCTGGATCTATCTTGTTTTGG |
| *sugE1*-QF | CGATTGCAGTTGCCTGTATG |
| *sugE1*-QR | AACGTATCAAGGAGGCGGTA |
| *sugE2*-QF | GTGATAACGCCCAGGATGAT |
| *sugE2*-QR | GTCGCAGTAGCCGTCTCTTT |
| *invH*-QF | GAACGCATGTATTGTGGAT |
| *invH*-QR | GTATGTGCCGTCAGACCTT |
| *invF*-QF | GAGAATGCTGGGAGAAGAC |
| *invF*-QR | AAATGTGAAGGCGATGAGT |
| *invG*-QF | GCAATCTGATACTACCACCTC |
| *invG*-QR | AAACGGAATACTTTGGACAG |
| *invE*-QF | CGCTGACCTACTGTTTGTG |
| *invE*-QR | GTGAAGAGGGTATGGCTTT |
| *invA*-QF | ATGTTATTCGCAAAGGGAT |
| *invA*-QR | ATCGACAGACGTAAGGAGG |
| *spaK*-QF | TATCGCTGAATTAGTTCGTT |
| *spaK*-QR | TCCATACATCATCGTCCTT |
| *spaL*-QF | ACCAGCGAGGGAAGCATTA |
| *spaL*-QR | GGTGACCGTCAAGGATAGAGC |
| *spaO*-QF | GCTTTGTAATCGGTAGCAGTG |
| *spaO*-QR | ACGGGAAGTACGAATCAGG |
| *spaP*-QF | ATTGCTGCTTTCTATGTTTG |
| *spaP*-QR | CTTTGTCACGCTTTACC |
| *spaQ*-QF | GTAGGGTTATTCCAGACGG |
| *spaQ*-QR | CGCCATACCAGCCAGACAG |
| *sicA*-QF | AGAACGTGTTGCGGAAATG |
| *sicA*-QR | GCTTCATCCAGTCGTCCCT |
| *sipB*-QF | TCAGGTTTCCCAGGGTGAG |
| *sipB*-QR | ACATCCCATAATGCGGTTC |
| *sipC*-QF | CGTTGAACCAGGCTGATAG |
| *sipC*-QR | GCTCTGGGAAATACTACCG |
| *sipD*-QF | AAATCCACCAGGCACAGCA |
| *sipD*-QR | CGCTCCGCAGGTTCTCATT |
| *sipA*-QF | ACGCCACCAGTGATTCTCC |
| *sipA*-QR | TCGCTTCCGCTTTCTTTGT |
| *iacP*-QF | TGCCGTTGATGTTGATAGT |
| *iacP*-QR | ACACCCTGGACTCAAGACT |
| *iagB*-QF | CCCACAATGACAGAAATGC |
| *iagB*-QR | TCCGAACTACTTTACGCTAT |
| *hilA*-QF | GTGGGCAACCAGCACTAAC |
| *hilA*-QR | ACGCAGGAAATAACAGGAC |
| *hilD*-QF | CCAAGTCGTTGCGTCGGTAT |
| *hilD*-QR | TTGCTTTCGGAGCGGTAAA |
| *prgH*-QF | GCAGGCGTTACCTTATTCC |
| *prgH*-QR | CCCTTGAGCCAGTCATCTTT |
| *prgI*-QF | TGATAATCTACAAACGCAGGTA |
| *prgI-*QR | AACGGAAGTTCTGAATAATGG |
| *prgJ*-QF | GAGGACCCTAATCTGGTGA |
| *prgK*-QF | TGCCGATGTGGATTATGAC |
| *prgK*-QR | ATTTGACGATTTCGCCTTA |
| *orgA*-QF | ATTGAGGAGGCATTGAAGC |
| *orgA*-QR | GGACGGTATTGGGATATTTT |
| *gyrb*-QF | TGATTGCGGTGGTTTCCGTA |
| *gyrb*-QR | GACGACGATTTTCGCGTCAG |
| claudin3-QF | CCTAGGAACTGTCCAAGCCG |
| claudin3-QR | CCCGTTTCATGGTTTGCCTG |
| occludin-QF | CAGCCTTCTGCTTCATCG |
| occludin-QR | GTCGGGTTCACTCCCATTA |
| ZO-1-QF | GACCTTGAGCAGCCGTCATA |
| ZO-1-QR | CCGTAGGCGATGGTCATAGTT |
| β-actin-QF | AGCCATGTACGTAGCCATCC |
| β-actin-QR | CTCTCAGCTGTGGTGGTGAA |
